# Meconium androgens are correlated with ASD-related phenotypic traits in early childhood in a familial enriched risk cohort

**DOI:** 10.1186/s13229-020-00395-6

**Published:** 2020-11-23

**Authors:** Dina Terloyeva, Alexander J. Frey, Bo Y. Park, Elizabeth M. Kauffman, Leny Mathew, Anna Bostwick, Erika L. Varner, Brian K. Lee, Lisa A. Croen, Margaret D. Fallin, Irva Hertz-Picciotto, Craig J. Newschaffer, Kristen Lyall, Nathaniel W. Snyder

**Affiliations:** 1 AJ Drexel Autism Institute, Drexel University, 3020 Market St, Suite 560, Philadelphia, PA 19104 USA; 2 Department of Epidemiology and Biostatistics, Drexel University School of Public Health, 3215 Market Street, Philadelphia, PA 19104 USA; 3 Department of Public Health, California State University Fullerton, 800 N. State College Blvd., Fullerton, CA 92831 USA; 4 Autism Research Program, Kaiser Permanente Division of Research, 2000 Broadway, Oakland, CA 94612 USA; 5 Wendy Klag Center for Autism and Developmental Disabilities, Department of Mental Health, Johns Hopkins Bloomberg School of Public Health, 624 N. Broadway, HH 850, Baltimore, MD 21205 USA; 6 Department of Public Health Sciences, Medical Investigation of Neurodevelopmental Disorders (MIND) Institute, School of Medicine, University of California, Davis, Davis, USA; 7 College of Health and Human Development, Penn State, University Park, PA 16802 USA; 8 Department of Microbiology and Immunology, Center for Metabolic Disease Research, Temple University Lewis Katz School of Medicine, Philadelphia, PA 19140 USA

**Keywords:** Autism-related traits, Androgen, Meconium, Sibling, Sex difference, Prenatal exposure

## Abstract

**Background:**

Prenatal exposure to increased androgens has been suggested as a risk factor for autism spectrum disorder (ASD). This hypothesis has been examined by measurement of steroids in amniotic fluid, cord blood, saliva, and blood with mixed results.

**Methods:**

To provide an orthogonal measure of fetal exposure, this study used meconium, the first stool of a newborn, to measure prenatal androgen exposure from infants in the Early Autism Risk Longitudinal Investigation (EARLI). EARLI is a familial-enriched risk cohort that enrolled pregnant mothers who already had a child with an ASD diagnosis. In the younger child, we investigated the association between meconium unconjugated (u) and total (t) concentrations of major androgens testosterone (T), dehydroepiandrosterone (DHEA), and androstenedione (A4), and ASD-related traits at 12 and 36 months of age. Traits were measured at 12 months with Autism Observation Scale for Infants (AOSI) and at 36 months with total score on the Social Responsiveness Scale (SRS). One hundred and seventy children had meconium and AOSI, 140 had meconium and SRS, and 137 had meconium and both AOSI and SRS.

**Results:**

Separate robust linear regressions between each of the log-transformed androgens and log-transformed SRS scores revealed three-way interaction between sex of the child, sex of the proband, and testosterone concentration. In the adjusted analyses, t-T, u-A4, and u-DHEA (*P* ≤ 0.01) were positively associated with AOSI scores, while u-T (*P* = 0.004) and u-DHEA (*P* = 0.007) were positively associated with SRS total score among females with female probands (*n* = 10). Additionally, higher concentrations of u-T (*P* = 0.01) and t-T (*P* = 0.01) predicted higher SRS total score in males with male probands (*n* = 63).

Limitations

Since we explored three-way interactions, this resulted in a limited sample size for some analyses. This study was from an enriched-risk cohort which may limit generalizability, and this study used ASD-assessment scales as outcomes instead of diagnostic categories. Additionally, the novel use of meconium in this study limits the ability to compare the results in this cohort to others due to the paucity of research on meconium.

**Conclusions:**

This study supports the utility of meconium for studies of endogenous fetal metabolism and suggests the sex of older siblings with autism should be considered as a biological variable in relevant studies.

## Background

Autism spectrum disorder (ASD) has an estimated prevalence of 1 in 59 among 8-year-old children in the USA [1–3]. Within this population level estimate, there is a strong sex-dependent bias with a 4:1 male/female ratio, with some groups observing a closer to 2:1 male-to-female ratio [4, 5]. The mechanism underlying this sex difference remains unknown but is of interest in understanding the etiology of ASD. Critically, it is unknown whether female-specific protective factors, male-specific risk factors, or both drive this difference.

The strong sex differential in ASD prevalence also suggests that developmental processes of early sex-differentiation interact with etiological events driving developmental changes of ASD that manifest later in life. Indeed, multiple studies implicate the fetal development stage in the origin of this disorder and link genetic influences, neuroanatomical changes, and environmental influences such as steroid exposures to ASD outcome [3, 6–12]. Steroid hormones are crucial for sex-differentiation of the human fetus and development of the central nervous system in animals [13]; therefore, several studies have suggested a potential link between abnormal steroid hormone levels and ASD. Androgens, the sex steroids that bind to the androgen receptor, have received the most study. Previous studies measured androgen levels in either amniotic fluid or cord blood to estimate fetal exposure to androgens. In two separate studies, the same group found a positive correlation between amniotic fluid testosterone levels and autism assessment scales [14, 15]. Other studies using either amniotic fluid or cord blood reported mixed, but mostly null results [16–19].

To overcome the limitations of amniocentesis and cord blood, we used meconium, the first stool of a newborn, to estimate prenatal androgen exposure. Since formation of meconium is thought to start around the 12–13th week of gestation, meconium can be noninvasively collected within 72 h of the birth of the child, and meconium has the potential to capture cumulative prenatal exposure [20, 21]. Previous studies have utilized meconium samples to examine prenatal exposure to both exogenous and endogenous compounds [22–24], and we recently developed a robust method to quantify androgens in human meconium [21]. Sampling of meconium therefore avoids the invasive nature of amniocentesis and the restricted time window of sampling cord blood at birth.

This study investigated the association between levels of androgens, namely, testosterone (T) and its weakly androgenic precursors, dehydroepiandrosterone (DHEA) and androstenedione (A4), measured in meconium, and autistic traits at 12 and 36 months of age. Since these steroids exist in both unconjugated and glucuronide and/or sulfate-conjugated forms, and previous research has suggested glucuronidation may be different in autistic individuals [25], we performed analysis on both unconjugated (u) and total (t) forms that include both conjugated and unconjugated. Our study sample was drawn from the Early Autism Risk Longitudinal Investigation (EARLI), an enriched ASD risk pregnancy cohort where enrolled families had an older child (the study proband) with an ASD diagnosis [26]. Park et al. also analyzed samples from the EARLI cohort and did not find an association between prenatal androgens measured in cord blood and ASD phenotype; however, stratification by the proband’s sex revealed a positive relationship between testosterone levels and ASD phenotype among participants whose older, affected sibling was a female [18]. Based on this previous sub-group analysis, we examined effect modification by sex of the child and sex of the proband.

## Methods

### Study population

This study used data and biosamples from the Early Autism Risk Longitudinal Investigation (EARLI). EARLI design and study population have been reported elsewhere [26]. In short, the study included mothers of children with ASD diagnoses: autistic disorder, Asperger syndrome, or pervasive developmental disorder not otherwise specified, who were pregnant at the time of recruitment. Pregnant women were recruited by 28th week of pregnancy and followed closely until their child reached 3 years of age. In addition to having a biological child with ASD diagnosis, and being no more than 28 weeks pregnant, women had to be of at least 18 years of age, able to speak English or Spanish, and live within 2 h of a study site. Recruitment was performed by four participating study centers: Drexel/Children’s Hospital of Philadelphia, John’s Hopkins/Kennedy Krieger Institute, UC Davis, and Northern California Kaiser Permanente, which represented Southeast Pennsylvania, Northeast Maryland, and Northern California.

The study staff provided mothers with meconium collection kits prior to delivery and made arrangements with birth delivery staff at the hospitals and birth centers to assist with sample collection and temporary storage. Meconium was collected as soon as it was passed and was stored in the hospital/birth center or at home in the freezer until it was retrieved by the study staff. The study enrolled a total of 236 mothers with 8 mothers having twins, which resulted in 244 children in total. One of the twins in each twin pair was removed from the dataset at random resulting in 236 children. Of these 236 children, 62 lacked meconium, 29 lacked AOSI assessment at 12 months, and 65 lacked SRS assessment at 36 months resulting in analytic sample size of 137 participants (61 females, 76 males). One hundred and seventy children had steroids measurements with AOSI, while 140 children had steroids measurements and SRS; therefore, we performed parallel analyses among children who had androgen measurements and AOSI score to ensure that the results in the analytic sample are consistent with the results in the sample with any available data. A flowchart of included and excluded sample sizes is included in Additional file 1: Fig. 1S.

**Fig. 1:**
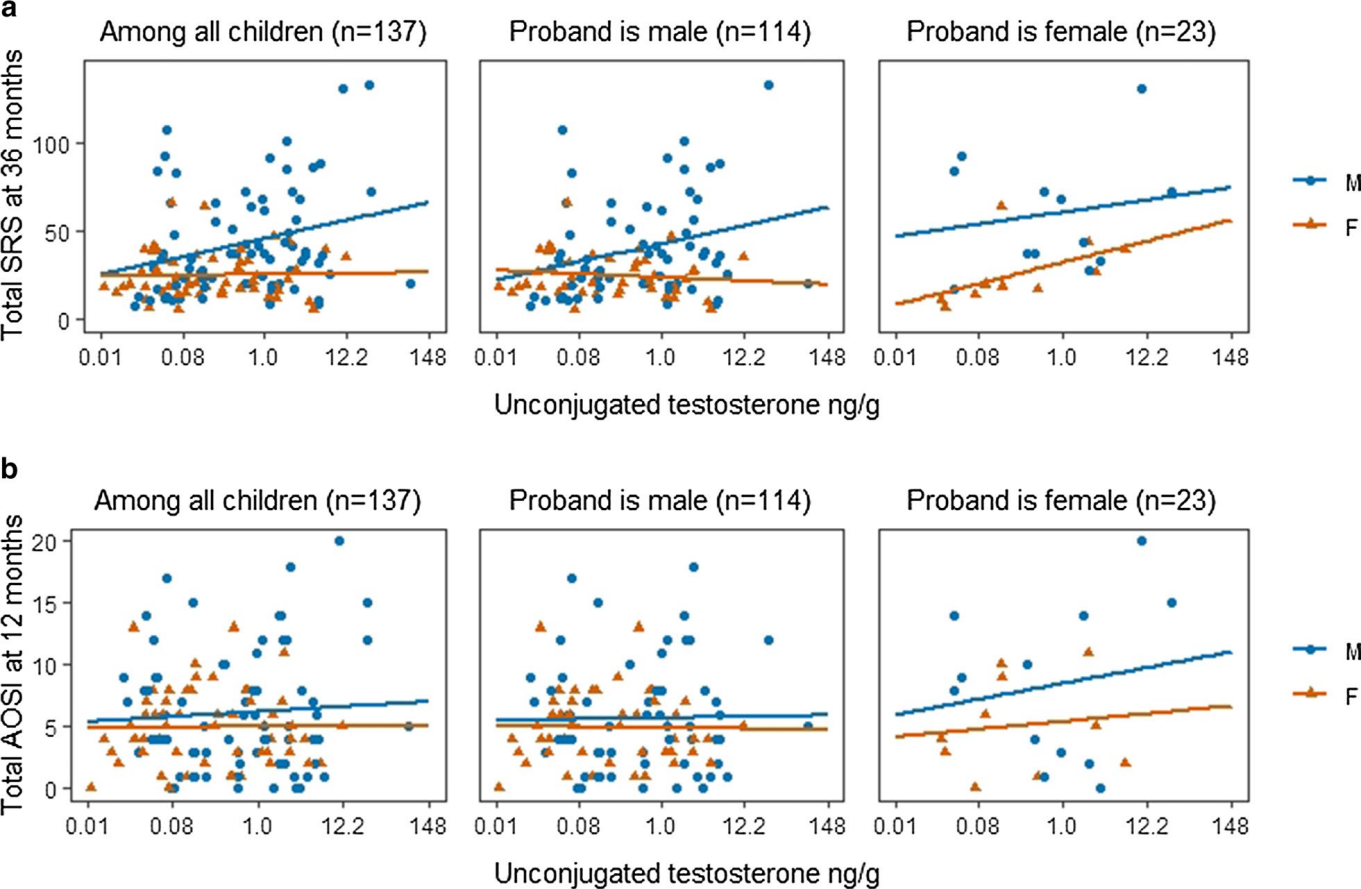
Relationship between meconium unconjugated testosterone (T) and 12- and 36-month outcomes by sex and further stratified by proband’s sex. **a** Unconjugated testosterone levels versus score on the social responsiveness scale (SRS). **b** Autism Observation Scale for Infants (AOSI)

### Laboratory methods

Meconium androgens were quantified by liquid chromatography–high resolution mass spectrometry (LC–MS/HRMS) as previously developed and validated [27] and then extended to meconium analysis [21]. Notably, this previous work indicates that over the timescales relevant to home collection androgens were stable in meconium. Briefly, both unconjugated (u) and total (t) (unconjugated plus conjugated) androgen levels were measured. T, A4, DHEA and internal standards (^13^C_3_-T, ^13^C_3_-A4, and ^2^H_5_-DHEA) were extracted from approximately 50 mg of each meconium sample by methanol, hexane/dicholoromethane (3:2), followed by derivatization with Girard P reagent and LC–MS/HRMS analysis as previously described. Limit of Quantification (LOQ) was defined as 10 times the lowest point on the calibration curve. LOQ was 2.4 ng/g for u-T, t-T, u-A4, and t-A4, and no samples fell below LOQ for DHEA (12 ng/g). Measurements of T fell below the limit of quantification defined at 2.4 ng/g for 7 participants (3 boys and 4 girls). For these participants, we used values of T replaced by $\frac{LOQ}{{\surd 2}}$.

### Outcome assessment

Outcomes included autistic traits measured at 12 months with the Autism Observation Scale for Infants (AOSI) [28] and at 36 months with the caregiver reported Social Responsiveness Scale (SRS), pre-school version [29]. AOSI is a semi-structured direct examiner-administered observational measure intended to capture early signs of autism in children aged 6–18 months. AOSI contains 18 items representing domains of behavior such as emotional responses, eye contact, visual tracking, social interest, motor and sensory behavior. Of these 18 items, 14 are assigned the score from 0 to 3, where 0 represents typical behavior and 3 represents a lack of behavior. The other four items, which include assessment of eye contact and atypical motor and sensory behavior, are rated on a scale of 0 to 2. Total score on the AOSI scale demonstrated high interrater reliability (unweighted kappa = 0.93) and acceptable test–retest reliability (intra-class correlation = 0.61) at 12 months with a score of 9 and greater was reported to be predictive of ASD diagnosis at 36 months [28]. EARLI study clinicians engaged in regular exercises to maintain cross-site reliability in AOSI administration as described previously [26].

SRS is a 65-item questionnaire completed by a caregiver at 36 months of age. Items on the SRS mainly assess reciprocal social behavior (RCB), and whether restricted or stereotypical behaviors and communicative deficits impact the child’s ability for RCB [30, 31]. Each item on the scale is assigned a score from 0 to 3, where 0 represents “never true” and 3 represents “almost always true”. The resulting total raw score ranges from 0 to 180, and higher scores indicate more severe social deficit. SRS has good psychometric properties and performs well among the general population of children and in children with older ASD-affected siblings [30–32]. Validation of the scale against the Autism Diagnostic Interview-Revised (ADI-R) showed high correlation between SRS scores and ADI-R scores [30].

### Covariates

Variables previously associated with ASD-phenotype were assessed for potential confounding: maternal age, race, ethnicity, education, income, gestational age, interpregnancy interval, number of previous pregnancies, and caesarian delivery. Assessment of covariates was performed in two steps. At first step, tests for independence were performed between each of the covariates and both exposures and outcomes before the decision was made to include them into the multivariable model. If both exposures and each of the outcomes were different between different levels of covariates irrespective of the *P* value, they were carried on to the second step. If the covariate was continuously measured, the correlation coefficient with both the exposure and the outcome had to be greater than 0.2 irrespective of the *P* value. As the second step, due to a limited sample size, covariates were incorporated into the model one at a time and change in the effect of the exposure on the outcome was measured. If this change was greater than 20%, the covariate was left in the model. Since sex of the child and sex of the proband were treated as effect modifiers, we performed stratified analysis of the effect of each androgen on SRS and AOSI: separate models were fit among female children with female probands, female children with male probands, male children with male probands, male children with female probands. In such cases when there were insufficient number of people in a level of a covariate, for example only one person of Hispanic ethnicity among girls with female probands, this covariate was not included in the model to avoid overfitting and inflating standard errors of the model coefficients. In cases when removing a covariate from the model resulted in more than 20% change in the effect estimate where the effect estimate in both models (with and without this covariate) was still close to zero and null according to the Wald’s t test (such as slope = 0.04 vs 0.02, with high *P* value), we removed the covariate from the model. In cases where two potential covariates (maternal and gestational age) did not qualify for inclusion into the multiple regression, they were still included to facilitate comparability with earlier published results on the same cohort [18]. Thus, final models in the entire sample were adjusted for maternal age, gestational age, and sex of the child. Final models stratified by proband’s sex were adjusted likewise: for maternal age, gestational age, and sex of the child. Models stratified by child’s sex, as well as models stratified by child’s sex and proband’s sex, were adjusted by maternal and gestational age.

### Statistical analyses

Natural log transformation was used to transform the distribution for symmetry of the meconium androgen levels. Both AOSI and SRS was positively skewed; therefore, we used natural log transformation of AOSI + 1 scores and SRS raw total score, respectively.

Separate robust linear regression models using multi-stage (MM) estimation [33] were fit to assess the association between each of the ln-transformed androgens and ln-transformed AOSI + 1 and SRS scores. Since sex of the child and proband’s sex were hypothesized to be effect modifiers, we included a three-way interaction term as well as all lower-level interactions between the variables contained in the three-way term in all the models. In particular, we included four interaction terms in the models: (1) between an androgen and sex of the child, (2) between an androgen and proband’s sex, (3) between sex of the child and proband’s sex, (4) and between an androgen, sex of the child, and proband’s sex. We then conducted exploratory analyses using separate robust regression models within each stratum defined by child’s sex and sex of the proband: female children with male probands (*n* = 51), female children with female probands (*n* = 10), male children with male probands (*n* = 63), and male children with female probands (*n* = 13). Additionally, we fit similar models with untransformed outcomes: total AOSI and total SRS, in order to facilitate more intuitive interpretations. In later sections, we discuss the results of the models with untransformed outcomes only if respective results from the models with ln-transformed outcomes were significant at 0.05 level.

Detailed model diagnostics were performed on every model, which included visual and quantitative analysis of residuals, identification of points with high leverage, and high influence. Multicollinearity of the multiple regression models was assessed with variance inflation factor using a threshold of 10, and heteroscedasticity was assessed by plotting residuals versus predicted values. None of the computed regression models met that threshold or displayed heteroscedasticity.

All analyses were performed in SAS version 9.4, and all plots were made in RStudio version 1.0.143. All study participants or their parents/guardians gave their informed consent under the EARLI study approved by the Institutional Review Board of Drexel University.

## Results

### Sample characteristics

Overall and sex-stratified characteristics of the 137 participants (61 females, 76 males) with all available data are shown in Table 1. Similar to the descriptive results presented by Park et al. [18] but using a slightly different analytic sample due to availability of cord blood versus meconium samples, there were no sex differences in geometric mean of AOSI score at 12 months: geometric mean (GM) = 5.4, geometric standard deviation (GSD) = 2.2 among males compared to GM = 4.9, GSD = 1.9 among females (*P* = 0.46). However, boys had on average higher SRS scores than girls: GM = 34.4 (GSD = 2.0), GM = 22.4 (GSD = 1.7, *P* < 0.0001). Male and female participants had similar distributions of maternal age, gestational age, inter-pregnancy interval, number of pregnancies, proportion of children delivered by cesarean section, maternal race and ethnicity, maternal education, and sex of the proband. However, there were differences in income: girls’ families were more likely to report lower income compared to boys’ families within the middle brackets of income (*P* = 0.03).

**Table 1:** Study characteristics by sex of the child

Characteristic	Total		Female		Male		*P*
	Mean	SD	Mean	SD	Mean	SD	
Maternal age	34.5	4.5	34.4	4.5	34.6	4.5	0.84
Gestational age at delivery (weeks)	39.3	1.7	39.3	1.8	39.3	1.5	0.96
Interpregnancy interval (days)	1671	968	1737	880	1617	1038	0.47
Total number of pregnancies	3.7	1.6	3.5	1.4	3.9	1.7	0.16
Androgen levels							
ln(u-T)	-1.2	2.1	-1.7	2.0	-0.8	2.1	0.0083
ln(t-T)	3.1	1.6	2.8	1.6	3.4	1.5	0.018
ln(u-A4)	2.9	0.6	2.9	0.6	2.8	0.5	0.30
ln(t-A4)	5.3	0.6	5.4	0.7	5.2	0.5	0.17
ln(u-DHEA)	2.4	0.8	2.5	0.8	2.3	0.7	0.051
ln(t-DHEA)	7.2	1.3	7.2	1.4	7.2	1.3	0.71
Outcome scores							
Total AOSI score^a^	5.2	2.1	4.9	1.9	5.4	2.2	0.46
Total SRS score^a^	28.4	2.0	22.4	1.7	34.4	2.0	< 0.0001

	Mean (median)	SD (IQR)	Mean (median)	SD (IQR)	Mean (median)	SD (IQR)	
Total AOSI score	5.5 (5)	4.1 (3–8)	4.8 (4)	3.1 (3–7)	6.0 (5)	4.8 (2.5–8.5)	0.33^b^
Total SRS score	35.7 (28)	25.8 (18–42)	25.6 (22)	13.2 (16–33)	43.8 (35)	30.2 (21–65)	< 0.001^b^

	%	*n*	%	*n*	%	*n*	
Cesarean delivery							
Yes	35.3	48	38.3	23	32.9	25	0.51
Maternal race							
White	66.9	89	61.0	36	71.6	53	0.50
Black	9.8	13	13.6	8	6.8	5	
Asian	12.0	16	13.6	8	10.8	8	
Other	11.3	15	11.9	7	10.8	8	
Maternal ethnicity							
Hispanic	17.5	24	16.4	10	18.4	14	0.76
Maternal education							
< 9th	3.7	5	4.9	3	2.7	2	0.94
High school graduate	25.9	35	26.2	16	25.7	19	
College	40.7	55	39.3	24	41.9	31	
Graduate/professional degree	29.6	40	29.5	18	29.7	22	
Income							
< $29,999	8.4	11	6.9	4	9.6	7	0.03
$30,000–49,999	16.8	22	25.9	15	9.6	7	
$50,000–74,999	15.3	20	15.5	9	15.1	11	
$75,999–99,999	16.0	21	6.9	4	23.3	17	
$100,000+	43.5	57	44.8	26	42.5	31	
Sex of the proband							
Female	16.8	23	16.4	10	17.1	13	0.91
Male	83.2	114	83.6	51	82.9	63	

^a^Geometric mean and geometric standard deviation

^b^Two-sided *P* value for Mann–Whitney *U* test

AOSI in the analytic sample was correlated with SRS score [Spearman correlation coefficient $\rho$ = 0.18 (*P* = 0.038)]. When stratified by sex, correlation was slightly higher among boys [Spearman $\rho$ = 0.29 (*P* = 0.012)] and was null among girls [Spearman $\rho$ = − 0.02 (*P* = 0.88)].

### Meconium androgen measurements

Levels of u-T and u-A4 were positively correlated in the entire sample, as well as among male and female participants (Table 2). u-T was positively correlated with u-DHEA among all participants and among girls. Similarly, u-A4 and u-DHEA were positively correlated with each other in the entire sample and among girls. Levels of total androgens were correlated with each other among all children, except for t-A4 with t-DHEA. In sex-stratified analysis, total androgens were positively correlated with each other, except for t-T with t-A4 and t-A4 with t-DHEA, which were not correlated among girls.

**Table 2:** Spearman rank correlation of unconjugated and total androgens in meconium

	All subjects (*n* = 137)			Male (*n* = 76)			Female (*n* = 61)		
	T-A4	A4-DHEA	DHEA-T	T-A4	A4-DHEA	DHEA-T	T-A4	A4-DHEA	DHEA-T
Unconjugated	0.33**	0.31**	0.17*	0.23*	0.15	0.09	0.50**	0.50**	0.39*
Total	0.27*	0.16	0.45**	0.40*	0.26*	0.47**	0.18	0.02	0.45*

*P value < 0.05

**P value < 0.0001

Both u-T and t-T were higher in males: median u-T = 0.57 ng/g (IQR: 0.07–2.27) among boys compared to 0.14 ng/g (IQR: 0.04–1.80) among girls (*P* = 0.01) and median total T = 31.97 ng/g (IQR: 10.00–115.67) among boys compared to 13.21 ng/g (IQR: 4.99–66.55) among girls (*P* = 0.02). Levels of meconium u-DHEA were higher for girls (*P* = 0.04): median = 10.19 ng/g (IQR: 7.86–17.96), compared to boys: median = 8.68 ng/g (IQR: 6.58–12.49). There were no significant differences between u- or t-A4, and t-DHEA between males and females. All of these differences mirror the findings within all participants with meconium samples in EARLI as previously reported [21].

### Associations of androgen levels and ASD-quantitative phenotype at 12 and 36 months: results of correlation analysis

There was no observed correlation between any of the ln-transformed androgens and 12-month AOSI scores in the full sample; however, after stratification by sex of the younger sibling, a weak negative correlation with t-A4 was seen among females (Spearman $\rho$ = − 0.32, *P* = 0.013) (Additional file 1: Table 1S). Further stratification by proband’s sex revealed stronger positive, marginally significant correlation with t-DHEA ($\rho$ = 0.54, *P* = 0.058) among males with female probands. Among females with male probands weak negative correlation with t-A4 ($\rho$ = − 0.31, *P* = 0.03) and weak negative correlation with u-DHEA ($\rho$ = − 0.32, *P* = 0.02) was observed.

Meanwhile, both ln-transformed u-T (Fig. 1) and t-T (Additional file 1: Fig. 2S) were positively associated with SRS in the entire sample ($\rho$ = 0.21, *P* = 0.02 and $\rho$ = 0.18, *P* = 0.03, respectively) (Additional file 1: Table 2S). This relationship persisted among males, but not among females. Further stratification by proband’s sex revealed weak positive correlation among males with male probands ($\rho$ = 0.24, *P* = 0.06 and $\rho$ = 0.26, *P* = 0.04 respectively). Negative correlation between ln-transformed t-T and SRS was seen among females with male probands ($\rho$ = − 0.30, *P* = 0.03). Among females with female probands strong positive correlation of SRS with ln-transformed u-T ($\rho$ = 0.70, *P* = 0.03), t-T ($\rho$ = 0.82, *P* = 0.004), u-A4 ($\rho$ = 0.67, *P* = 0.03), and u-DHEA ($\rho$ = 0.77, *P* = 0.01).

**Fig. 2:**
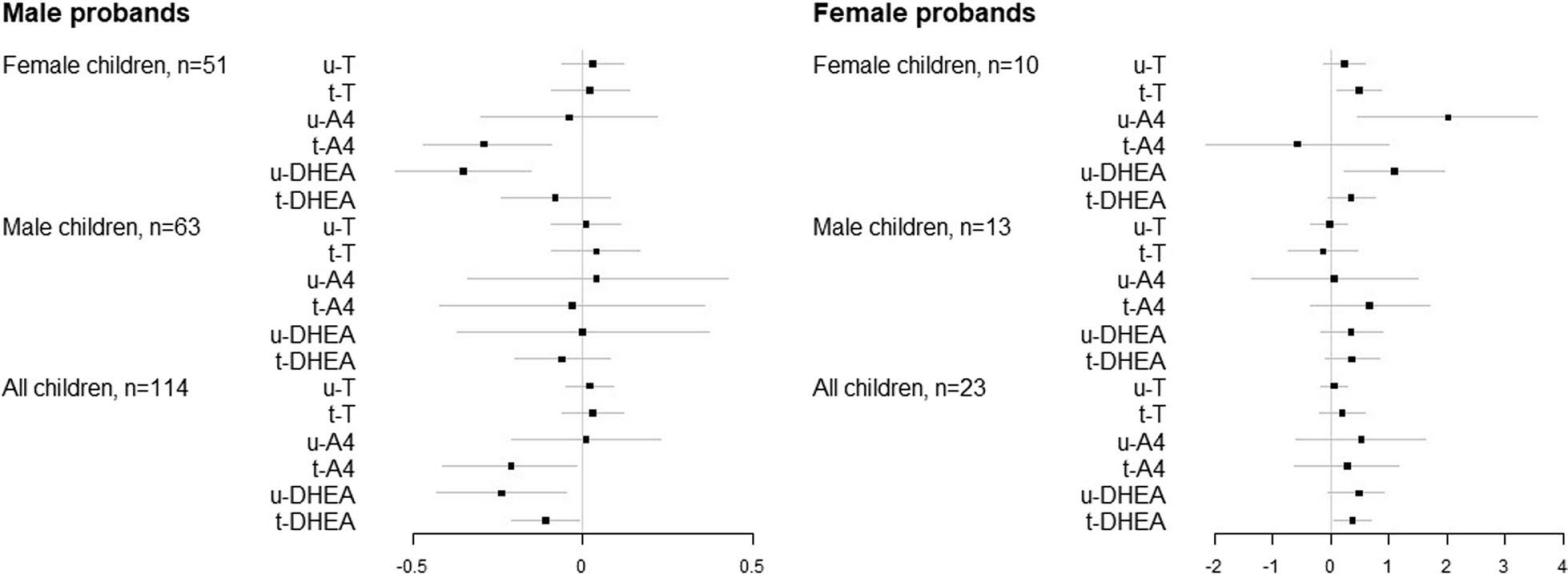
Coefficients and associated 95% Confidence Intervals (CIs) of the ln-transformed androgens from the adjusted robust linear regression model predicting ln-transformed AOSI score. The models were adjusted for maternal and gestational age

### Results of unadjusted and adjusted robust linear regression models with AOSI score

The results of robust linear regression models between androgen levels and AOSI score at 12 months are shown in Fig. 2 and Additional file 1: Tables 1S (log–log model) and Additional file 1: 2S (ln(androgen) and total AOSI). Scatterplots for each other androgen measured and total SRS or total AOSI are shown in Additional file 1: Figs. 2S–6S. In the full sample, there was no observed association between any of the androgen levels and AOSI. When we stratified by sex of the child, we observed inverse association between u-DHEA and t-A4 and AOSI among female children: in the unadjusted analysis 25% increase in the u-DHEA predicted 4.2% ($1.25^{ - 0.19} = 0.958,\;P = 0.065$) and 6.1% ($1.25^{ - 0.28} = 0.949,\;P = 0.006$) decrease in the AOSI score in the unadjusted analysis and adjusted analyses, respectively, while 25% increase in t-A4 predicted 5.6% $\left( {1.25^{ - 0.26} = 0.944,\;P = 0.01} \right)$ and 6.7% $\left( {1.25^{ - 0.31} = 0.933,\;P = 0.002} \right)$ decrease in AOSI in the unadjusted and adjusted analyses, respectively.

Models with untransformed AOSI score provide a more intuitive interpretation (Additional file 1: Table S2): one unit increase in natural logarithm of u-DHEA predicted 1.01-unit decrease in AOSI (or 0.24 standard deviation of standardized AOSI) in female children in the adjusted model. In the same group, one unit increase in natural logarithm of t-A4 was associated with 1.37 decrease in raw AOSI (or 0.33 standard deviation of standardized AOSI). There was no association between any of the androgens and AOSI among boys.

When stratified by proband’s sex, u-DHEA, t-A4, and t-DHEA were associated with AOSI among children with male probands: 25% increase in u-DHEA predicted 4.2% decrease in AOSI ($1.25^{ - 0.19} = 0.958,\;P = 0.03$) in the unadjusted model, and 5.2% decrease in the model adjusted for maternal age, gestational age, and child’s sex ($1.25^{ - 0.24} = 0.948,\;P = 0.08$). Additionally, 25% increase in t-A4 was associated with 4.2% ($1.25^{ - 0.19} = 0.958,\;P = 0.05$) and 4.6% ($1.25^{ - 0.21} = 0.954,\;P = 0.031$) decrease in AOSI in the unadjusted and adjusted models, respectively, in this group. t-DHEA was not associated with AOSI in the unadjusted analysis (${\text{slope }} = { } - 0.07,\;P = 0.16$); however, after adjusting for maternal age, gestational age, and child’s sex, 25% increase in t-DHEA predicted 2.4% decrease in AOSI in children with male probands ($1.25^{ - 0.11} = 0.976,\;P = 0.037$).

The same adjusted models with AOSI outcome showed that one unit increase in natural logarithm of u-DHEA and t-A4 was associated with almost one-unit decrease in AOSI score (or 0.24 standard deviation of standardized AOSI), while one unit increase in natural logarithm of t-DHEA predicted 0.32 decrease in AOSI (0.08 standard deviation of standardized AOSI) in children with male probands.

Among children with female probands, u-DHEA and t-DHEA were positively associated with AOSI. Although neither of these androgens were statistically significantly associated with AOSI in the unadjusted analysis, when adjusted for maternal age, gestational age, and child’s sex, 25% increase in u-DHEA predicted 11.6% increase in AOSI ($1.25^{0.49} = 1.116,\;P = 0.02$), while 25% increase in t-DHEA predicted 8.6% increase in AOSI ($1.25^{0.37} = 1.086,\;P = 0.018$).

According to the adjusted models with untransformed AOSI in the same group, one unit increase in natural logarithm of u-DHEA was associated with 4.04-unit increase in AOSI (or 0.97 standard deviation of standardized AOSI), while one-unit increase in natural logarithm of t-DHEA predicted 1.85 unit increase in AOSI score (or 0.45 standard deviation of standardized AOSI score).

When stratified by proband’s sex and sex of the child, although no association between androgens and AOSI among females with older affected female siblings was evident in the unadjusted models, positive association was found between t-T, u-A4, and u-DHEA and AOSI in this group after adjusting for maternal and gestational age. In fact, the models predicted that with every 25% increase in the levels of total T, unconjugated A4, and unconjugated DHEA, AOSI would increase by 11% ($1.25^{0.48} = 1.11,\;P = 0.014$), 57% ($1.25^{2.02} = 1.57,\;P = 0.01$), and 28% ($1.25^{1.09} = 1.28, \;P = 0.01$), respectively. Same adjusted models but with untransformed outcome showed that AOSI will increase by 1.62 (or 0.39 standard deviation of standardized AOSI) with one unit increase in natural logarithm of total T, by 7 (1.69 standard deviations of standardized AOSI) with every unit increase in natural logarithm of unconjugated A4, and by 5.06 (1.22 standard deviations of standardized AOSI) with every unit increase in natural logarithm of unconjugated DHEA (Additional file 1: Table 2S).

Inverse associations between levels of some androgens and quantitative ASD phenotype were found among females with older affected male siblings. Specifically, AOSI was negatively associated with u-DHEA: the unadjusted model predicted 6% decrease in AOSI with 25% increase in unconjugated DHEA ($1.25^{ - 0.26} = 0.94,\;P = 0.01$), while adjusted model predicted 8% decrease in AOSI with 25% increase in DHEA ($1.25^{ - 0.35} = 0.92,\;P = 0.001$). Similar adjusted model but with untransformed AOSI predicted 1.13-point decrease in AOSI (0.27 standard deviation of standardized AOSI) with one unit increase in ln-transformed DHEA. Meanwhile, 25% increase in the levels of t-A4 predicted on average decrease of 5% and 6% in AOSI, according to unadjusted and adjusted models respectively ($1.25^{ - 0.25} = 0.95,\;P = 0.01$, and $1.25^{ - 0.29} = 0.94,\;P = 0.003$). According to the adjusted model with total AOSI as an outcome, one unit increase in ln-transformed t-A4 will result in 1.19-point decrease in AOSI, which is 0.29 standard deviation of standardized AOSI.

In the group of male children with older affected male siblings, the regression analyses did not find associations between any of the androgens and the AOSI score. Similarly, no associations were found between any of the androgen concentrations and AOSI among males with female probands.

### Results of unadjusted and adjusted robust linear regression models with SRS score

**Fig. 3:**
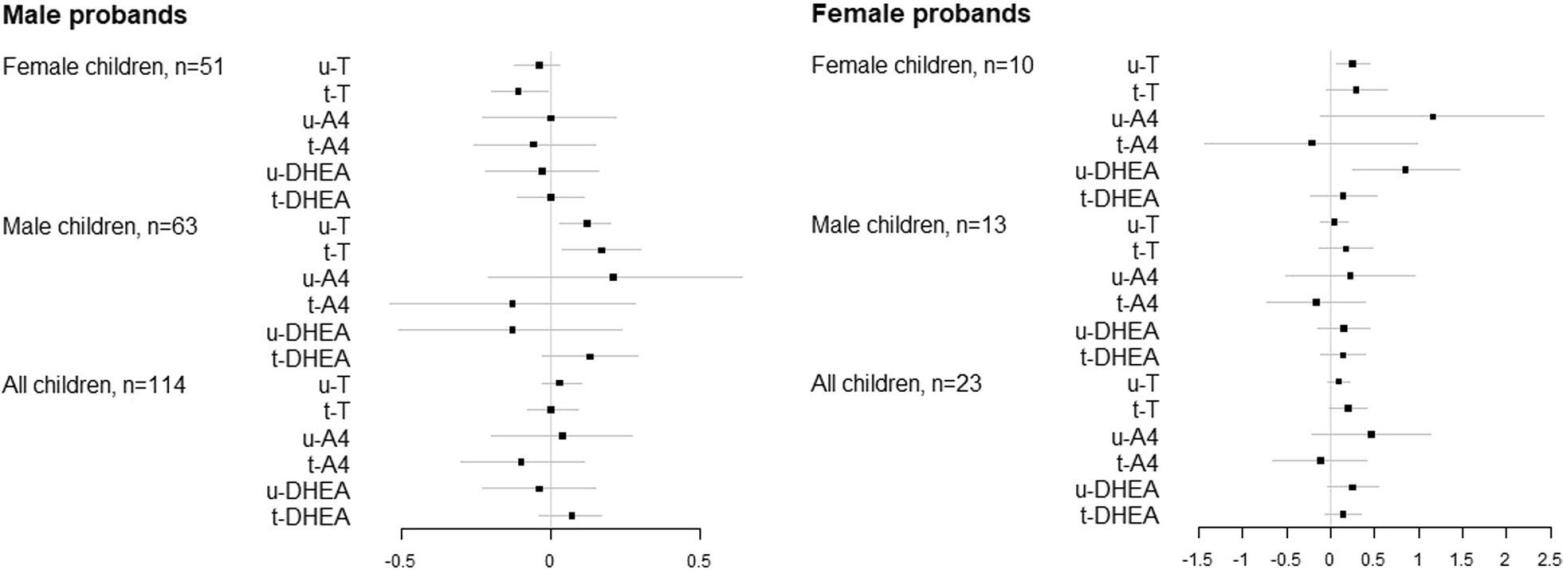
Coefficients and associated 95% Confidence Intervals (CIs) of the ln-transformed androgens from the adjusted robust linear regression models predicting ln-transformed SRS score. The models were adjusted for maternal and gestational age

The results of robust linear regression models between androgen levels and SRS score at 12 months are shown in Fig. 3 and Additional file 1: Tables 3S (log–log model) and Additional file 1: 4S (ln(androgen) and total SRS).

In the sample of all children u-T and t-DHEA were weakly associated with SRS: 25% increase in both u-T and t-DHEA levels predicted almost 2.0% increase in SRS in both adjusted and unadjusted models ($1.25^{0.08} = 1.018,\;P = 0.01$, for u-T, and $1.25^{0.08} = 1.018,\;P = 0.08$ for t-DHEA in the unadjusted model, and $1.25^{0.09} = 1.02,\;P = 0.049$ for t-DHEA in the adjusted model). In the adjusted models with SRS, one unit increase in the natural logarithm of u-T was associated with 1.31-unit increase in SRS score (0.05 standard deviation of standardized SRS), while one-unit increase in natural logarithm of t-DHEA was associated with 1.78-unit increase in SRS (0.07 standard deviation of standardized SRS).

When stratified by sex of the child, none of the androgens were statistically significantly associated with SRS among female children. However, among boys 25% increase in u-T, t-T, and t-DHEA predicted 2.5% ($1.25^{0.11} = 1.025,\;P = 0.01$), 3.6% ($1.25^{0.16} = 1.036,\;P = 0.01)$, and 3.6% ($1.25^{0.16} = 1.036,\;P = 0.03)$ increase in SRS in the unadjusted models, respectively. In the adjusted models, 25% increase in u-T, t-T, and DHEA predicted 2.5% ($1.25^{0.11} = 1.025,\;P = 0.02$), 3.6% ($1.25^{0.16} = 1.036,\;P = 0.01)$, and 3.4% ($1.25^{0.15} = 1.034,\;P = 0.048$) increase in SRS score, respectively. According to the same adjusted models with raw SRS, one unit increase in natural logarithm of u-T, t-T, and t-DHEA was associated with 3.11, 4.65, and 5.23-unit increase in SRS, respectively (0.12, 0.18, and 0.20 standard deviation of standardized SRS).

When stratified by sex of the proband, in children with female probands, 25% increase in u-T was associated with 3.6% increase in SRS in the unadjusted model, ($1.25^{0.16} = 1.036,\;P = 0.036$); however, after adjustment for maternal and gestation age and sex of the child, the relationship was no longer statistically significant (slope = 0.09, $P = 0.17$). Similarly, in children with female probands 25% increase in t-T predicted 6.2% increase in SRS ($1.25^{0.27} = 1.062,\;P = 0.014$); however, the relationship was only marginally statistically significant after adjustment for potential confounding factors: 25% increase in t-T was associated with 4.6% increase in SRS ($1.25^{0.20} = 1.046$, $P = 0.07$). In contrast, none of the androgens were associated with SRS in children with male probands in unadjusted or adjusted models.

When stratified by sex of the child and sex of the proband, in the group of female children with older affected female siblings, the robust regression models revealed positive associations between u-T and SRS in both unadjusted and adjusted analyses (Fig. 3 and Additional file 1: Tables 3S and 4S): 6% and 5% increase in SRS with 25% increase in u-T, respectively ($1.25^{0.26} ,\;P < 0.001$, and $1.25^{0.22} ,\;P = 0.004$). Similar adjusted model with untransformed SRS showed that SRS will increase by 5.76 points (0.22 standard deviation of standardized SRS) with every unit increase in ln-transformed u-T. In the same group, positive association between t-T and SRS was present in the unadjusted model (7% increase in SRS with 25% increase in t-T: $1.25^{0.31} ,\;P = 0.003$), but lost statistical significance in the multivariable model (7% increase in SRS with 25% increase in t-T: $1.25^{0.31} ,\;P = 0.10$). In the adjusted model with untransformed SRS, one unit increase in natural logarithm of t-T predicted 7.3-point increase in SRS (0.28 standard deviation of standardized SRS). Similarly, u-A4 was positively associated with SRS in girls with female probands in the unadjusted analysis predicting 36% increase in SRS with 25% increase in the androgen level ($1.25^{1.37} = 1.36,\;P = 0.002$); however, the relationship was only marginally statistically significant in the adjusted analysis ($1.25^{1.16} = 1.30, P = 0.07$). The adjusted model with total SRS as an outcome predicted that one unit increase in natural logarithm of u-A4 will result in 29.71-point increase in SRS (or 1.15 standard deviations of standardized SRS). In the same group, u-DHEA was associated with SRS in both unadjusted and adjusted analyses: the models predicted 23% and 21% increase in SRS with 25% increase in u-DHEA, respectively ($1.25^{0.92} = 1.23,\;P = 0.001$, and $1.25^{0.85} = 1.21,\;P =$ 0.007). In the similar adjusted model but with untransformed outcome, one unit increase in natural logarithm of u-DHEA predicted 41.95-point increase in SRS (1.63 standard deviations of standardized SRS) (Additional file 1: Table 4S).

u-T and t-T were associated with SRS in both single and multiple regression models in the group of male children with older affected male siblings. Both unadjusted and adjusted models predicted 3% increase in SRS score with 25% increase in u-T ($1.25^{0.12} = 1.03,\;P = 0.0{2}$, and $P = 0.0{1}$, respectively), while 25% increase in t-T will result in 3 and 4% increase in SRS according to the unadjusted and adjusted models, respectively ($1.25^{0.15} = 1.03,\;P = 0.04$, and $1.25^{0.17} = 1.04,\;P = 0.01$). Adjusted models with untransformed SRS showed that one unit increase in natural logarithm of u-T will result in 3.66-point increase in SRS, which translates into 0.14 standard deviation of standardized SRS, while one unit increase in natural logarithm of t-T will result in 4.41-point increase in SRS (or 0.17 standard deviation of standardized SRS in this group).

Consistent with AOSI results in female children with older affected male siblings, SRS was negatively associated with total testosterone in this group: both unadjusted and adjusted models predicted an average 2% decrease in SRS with 25% increase in total T ($1.25^{ - 0.09} = 0.98,\;P = 0.06$, and $1.25^{ - 0.11} = 0.976,\;P = 0.02$, respectively). Meanwhile, adjusted model with untransformed SRS predicted 2.43 decrease in total SRS (0.09 standard deviation of standardized SRS) with one unit increase in natural logarithm of t-T. In contrast, we found no associations between any of the androgen concentrations and SRS among males with female probands.

## Discussion

This study investigated the correlations between meconium androgen concentrations and ASD traits, as measured by AOSI at 12 months and SRS at 36 months, among children whose older sibling was diagnosed with ASD. We built upon previous analysis by including stratification by proband sex and by including both unconjugated and conjugated forms of androgens in meconium.

When comparing both the 12 month AOSI scores and the 36 month SRS scores, the positive associations were observed more frequently between meconium androgens and SRS. Specifically, unconjugated T and total DHEA were positively associated with SRS among all children. This broadly agrees with studies on unconjugated T, amniotic fluid, and ASD-related traits during this time window [14, 34]. These positive associations are consistent with Auyeung and colleagues who reported the association between fetal T and ASD-assessment scales in the combined analyses as well as among boys and girls. However, our results are not consistent with Park et al., Whitehouse et al., and Jamnadass et al. who reported null results in both combined and sex-stratified analyses [16, 17, 19]. As we noted in the introduction, part of the discrepancy in these findings could be attributed to sampling of amniotic fluid versus cord blood across studies.

When stratified by sex of the child, unconjugated T, total T, and total DHEA were positively associated with SRS among male children, and when further stratified by proband’s sex, unconjugated and total T were positively associated with SRS among male children with male probands, but not female children with male probands, while positive association was observed between unconjugated T and SRS among female children with female probands but not among male children with female probands. The models with AOSI outcome revealed positive associations, when proband was female: total DHEA was associated with AOSI among all children with female probands, while total T and unconjugated DHEA were associated with AOSI among female children with female probands. The trend of positive correlations between T and SRS in male children is again consistent with literature on amniotic fluid androgens. The trend of positive associations occurring when the proband is a female agrees with a previous study completed with this same EARLI cohort where the strata with a female proband was observed in a secondary analysis to be the only strata with a detectable association between cord blood unconjugated testosterone and SRS [18]. In meconium from this female proband stratum, associations became apparent and statistically significant that often were not seen in the entire sample or in the strata defined only by sex. Interestingly, robust linear regression models with SRS among female children with female probands frequently estimated high $r^{2}$ values even in the crude analysis, indicating that the exposure variable (meconium androgen levels) alone could explain a substantial amount of variability in the ASD-assessment scores in this group. When SRS was the outcome, we observed relatively strong, statistically significant associations with high $r^{2}$ in the adjusted and unadjusted analyses in the models with unconjugated T and unconjugated DHEA. While associations between SRS and unconjugated A4, total T, and total DHEA were not statistically significant in this group, they were positive with high $r^{2}$ values.

Furthermore, our study reported a series of negative relationships, all of which occurred in children whose older affected sibling was male. u-DHEA was negatively associated with AOSI in the sample of all female children. When further stratified by proband’s sex, this relationship persisted among female children with male probands but became positive in female children with female probands. Similarly, negative relationship between total A4 and AOSI was detected among female children; however, after stratification by proband’s sex it persisted only among female children with male probands. Total DHEA was negative associated with AOSI among children with male probands. Additionally, and counter to the trend in male children with male probands, total T was inversely associated with SRS in female children with male probands. The negative correlation we observed of T and SRS among females with a male proband has not been reported to our knowledge.

Effect modification of the association between androgens and ASD-phenotype by sex of the participants was shown in multiple studies , including the ones that demonstrated that girls are more susceptible to the effect of abnormal fetal testosterone levels than boys [35–39]. Effect modification of the association by probands’ sex was reported by Park et al. using the same EARLI study sample as this study [27], where unconjugated fetal testosterone measured in cord blood was associated with increase in both AOSI and SRS among siblings of ASD-affected females. In addition, there was a marginally significant association between A4 and SRS in the analysis stratified by proband’s sex and adjusted for child’s sex. Thus, within EARLI in both cord blood and meconium, we detect triple interaction between sex steroids, child’s sex, and proband’s sex where the interaction between androgens and sex is different by probands’ sex. The consistency of positive correlation across different androgens, different outcomes in this group, and different biosamples reflecting different windows of prenatal metabolism is worth highlighting. It is now important to replicate these findings using other cohorts, and future studies would benefit from including proband sex as a consideration in study design.

Finally, the analysis of both unconjugated and conjugated androgens in meconium introduces some possible alternative explanations of trends in the data. Since meconium is a unique fecal specimen and constitutes a compartment within the fetus that may have limited recycling back to fetal circulation [40], sequestration of androgens in the meconium may indicate less fetal exposure. Conjugation of steroids may increase this sequestration, explaining protective effects (negative correlations) in meconium androgens and ASD-related traits. Although adult fecal excretion of steroids is minor and predominated by conjugated steroids, possibly attributable to enterohepatic recycling, this pattern of clearance is neither true in pregnancy or in newborns [41]. Within the fetal compartment, where urinary clearance results in recycling, meconium becomes even more unique as a sequestered depot of bioactive molecules.

## Limitations

Our study had several limitations. Limited sample sizes, especially in the group of female children with female probands add substantial uncertainty to the findings. Many of the positive associations found among girls with female older affected siblings, as well as associations found in other strata could have been due to chance. Furthermore, this study analyzed the associations between six androgens and two outcomes in four strata, which presents a multiple testing problem. Since we were interested in attempting to replicate the sub-group analysis of Park et al., and our limited sample size for these levels of stratification, we did not use multiplicity correction. Therefore, the authors consider this study to be exploratory in nature and we warn the reader to consider the results with caution in the view of data scarcity and the compounded multiple testing problem induced by examining sex as a biological variable in the context of a familial enriched risk cohort.

Another limitation of this study is considerable variability of the meconium androgen measurements, even after ln-transformation of the exposure variables. Due to limited sample size, and the fact that since this was the first quantitative study of meconium androgens, we used robust regression models rather than removal of outliers where normal biological variability was unknown. Since this decision may have a strong effect on our results, we presented full scatterplots of the analyzed data to provide a comprehensive view of the reported relationships. The limited number of studies to date on endogenous metabolites in meconium limits our ability to compare the measures in this cohort versus others. Likewise, the physiological distinction between unconjugated and conjugated steroids in meconium is unclear. Although analyzing both forms of steroids inflated the potential for type I error via multiple comparisons, we thought this was necessitated by the fact that both pools of metabolites were consistently quantifiable within meconium. Similarly, because of the paucity of data on meconium, we did not analyze other steroids, including estrogens, in meconium. This may be important in understanding the correlations between fetal steroids and ASD outcomes have implicated maternal steroids and estrogens in ASD [42, 43]. Future work including validation of bioanalytical assay of other hormones and their stability in meconium is needed.

Additionally, this study used ASD-assessment scales as outcomes instead of diagnostic categories. While scales, such as AOSI or SRS, provide a continuous measure of ASD-related phenotype, hence increased power for the statistical analysis, the relationship between higher scores on these scales and ASD diagnoses is not unequivocal [44]. However, some studies showed that relationships between certain genetic and environmental exposures and ASD have similar magnitudes irrespective of using ASD diagnoses or continuous scale, suggesting that both higher scores on ASD-assessment scales and ASD diagnosis reflect a similar outcome [45, 46].

Finally, our findings must be considered within the constraints of the enriched-risk cohort design, meaning that the risk of ASD in this cohort was higher than in the general population by design. If the effect of fetal steroids on ASD phenotype is modified by genetic architecture enriched in this cohort, or severity by selection on the proband, the findings could be attenuated in the broader population of people without family history of ASD. Thus, replication of our findings in studies utilizing samples from a general population may provide evidence supporting generalizability of the results of this study.

## Conclusions

Using a prospective pregnancy cohort enriched for autism risk, we investigated prenatal androgen exposure measured from meconium as a risk factor for autism-related traits. Several meconium androgens were positively correlated with autism-related traits. In addition, we found a strong positive association between autism traits in the sub-group of individuals with an older female sibling with autism, extending a previous finding based on cord blood measures in the same cohort. This study supports the utility of meconium for studies of endogenous fetal metabolism and suggests the sex of the proband should be considered as a biological variable in relevant studies.

## Supplementary information

**Additional file 1.** Supplementary tables and figures.

## Data Availability

The datasets used and/or analyzed during the current study are available through the National Database for Autism Research (NDAR) under the Early Autism Risk Longitudinal Investigation (EARLI) Network (https://nda.nih.gov/edit_collection.html?id=1600).

## Abbreviations

EARLI: Early Autism Risk Longitudinal Investigation
ASD: Autism spectrum disorder
u-T: Unconjugated testosterone
u-DHEA: Unconjugated dehydroepiandrosterone
u-A4: Unconjugated androstenedione
t-T: Total testosterone
t-DHEA: Total dehydroepiandrosterone
t-A4: Total androstenedione
AOSI: Autism Observation Scale for Infants
SRS: Social Responsiveness Scale
LC–MS/HRMS: Liquid chromatography–high resolution mass spectrometry
LOQ: Limit of quantification
RCB: Reciprocal social behavior
ADI-R: Autism Diagnostic Interview-Revised
GM: Geometric mean
GSD: Geometric standard deviation
IQR: Interquartile range

## References

[1] Baio J (2018). Prevalence of autism spectrum disorder among children aged 8 years—autism and developmental disabilities monitoring network, 11 sites, United States, 2014. MMWR Surveill Summ.

[2] Weintraub K (2011). The prevalence puzzle: autism counts. Nature.

[3] Lyall K (2017). The changing epidemiology of autism spectrum disorders. Annu Rev Public Health.

[4] Boyle CA (2011). Trends in the prevalence of developmental disabilities in US children, 1997–2008. Pediatrics.

[5] Elsabbagh M (2012). Global prevalence of autism and other pervasive developmental disorders. Autism Res.

[6] Rodier PM, Ingram JL, Tisdale B, Nelson S, Romano J (1996). Embryological origin for autism: developmental anomalies of the cranial nerve motor nuclei. J Comp Neurol.

[7] Bauman ML, Kemper TL (2005). Neuroanatomic observations of the brain in autism: a review and future directions. Int J Dev Neurosci.

[8] Acosta MT, Pearl PL (2003). The neurobiology of autism: new pieces of the puzzle. Curr Neurol Neurosci Rep.

[9] Johnson MB (2009). Functional and evolutionary insights into human brain development through global transcriptome analysis. Neuron.

[10] Rice D, Barone S (2000). Critical periods of vulnerability for the developing nervous system: evidence from humans and animal models. Environ Health Perspect.

[11] Baron-Cohen S (2015). Elevated fetal steroidogenic activity in autism. Mol Psychiatry,.

[12] Knickmeyer RC, Baron-Cohen S (2006). Topical review: fetal testosterone and sex differences in typical social development and in autism. J Child Neurol.

[13] Peper JS, Koolschijn PC (2012). Sex steroids and the organization of the human brain. J Neurosci.

[14] Auyeung B (2012). Prenatal versus postnatal sex steroid hormone effects on autistic traits in children at 18 to 24 months of age. Mol Autism.

[15] Auyeung B (2009). Fetal testosterone and autistic traits. Br J Psychol.

[16] Jamnadass ES (2015). The perinatal androgen to estrogen ratio and autistic-like traits in the general population: a longitudinal pregnancy cohort study. J Neurodev Disord.

[17] Whitehouse AJ (2012). Perinatal testosterone exposure and autistic-like traits in the general population: a longitudinal pregnancy-cohort study. J Neurodev Disord.

[18] Park BY (2017). Umbilical cord blood androgen levels and ASD-related phenotypes at 12 and 36 months in an enriched risk cohort study. Mol Autism.

[19] Kung KT (2016). No relationship between prenatal androgen exposure and autistic traits: convergent evidence from studies of children with congenital adrenal hyperplasia and of amniotic testosterone concentrations in typically developing children. J Child Psychol Psychiatry.

[20] Park BY, Lee BK (2014). Use of meconium in perinatal epidemiology: potential benefits and pitfalls. Ann Epidemiol.

[21] Frey AJ (2017). Differences in testosterone and its precursors by sex of the offspring in meconium. J Steroid Biochem Mol Biol.

[22] Ostrea EM (2009). Combined analysis of prenatal (maternal hair and blood) and neonatal (infant hair, cord blood and meconium) matrices to detect fetal exposure to environmental pesticides. Environ Res.

[23] Kinsella RA, Francis FE (1971). Steroids and sterols in meconium. J Clin Endocrinol Metab.

[24] Gray TR (2009). Identification of prenatal amphetamines exposure by maternal interview and meconium toxicology in the Infant Development, Environment and Lifestyle (IDEAL) study. Ther Drug Monit.

[25] Stein TP, Schluter MD, Steer RA, Ming X (2013). Autism and phthalate metabolite glucuronidation. J Autism Dev Disord.

[26] Newschaffer CJ (2012). Infant siblings and the investigation of autism risk factors. J Neurodev disord.

[27] Frey AJ (2016). Validation of highly sensitive simultaneous targeted and untargeted analysis of keto-steroids by Girard P derivatization and stable isotope dilution-liquid chromatography-high resolution mass spectrometry. Steroids.

[28] Bryson SE (2008). The Autism Observation Scale for Infants: scale development and reliability data. J Autism Dev Disord.

[29] Constantino J, Gruber C (2005). Social responsive scale (SRS) manual.

[30] Constantino JN (2003). Validation of a brief quantitative measure of autistic traits: comparison of the social responsiveness scale with the autism diagnostic interview-revised. J Autism Dev Disord.

[31] Constantino JN (2006). Autistic social impairment in the siblings of children with pervasive developmental disorders. Am J Psychiatry.

[32] Constantino JN, Todd RD (2003). Autistic traits in the general population: a twin study. Arch Gen Psychiatry.

[33] Yohai VJ (1987). High breakdown-point and high efficiency robust estimates for regression. Ann Stat.

[34] Auyeung B, Taylor K, Hackett G, Baron-Cohen S (2010). Foetal testosterone and autistic traits in 18 to 24-month-old children. Mol Autism.

[35] Knickmeyer R (2006). Androgens and autistic traits: a study of individuals with congenital adrenal hyperplasia. Horm Behav.

[36] Hines M, Brook C, Conway GS (2004). Androgen and psychosexual development: core gender identity, sexual orientation and recalled childhood gender role behavior in women and men with congenital adrenal hyperplasia (CAH). J Sex Res.

[37] Hines M, Constantinescu M, Spencer D (2015). Early androgen exposure and human gender development. Biol Sex Differ.

[38] Hines M (2011). Gender development and the human brain. Annu Rev Neurosci.

[39] Pasterski V (2011). Prenatal hormones and childhood sex segregation: playmate and play style preferences in girls with congenital adrenal hyperplasia. Horm Behav.

[40] Barr DB, Bishop A, Needham LL (2007). Concentrations of xenobiotic chemicals in the maternal-fetal unit. Reprod Toxicol.

[41] Schiffer L (2019). Human steroid biosynthesis, metabolism and excretion are differentially reflected by serum and urine steroid metabolomes: a comprehensive review. J Steroid Biochem Mol Biol.

[42] Bilder DA (2019). Early second trimester maternal serum steroid-related biomarkers associated with autism spectrum disorder. J Autism Dev Disord.

[43] Baron-Cohen S (2019). Foetal oestrogens and autism. Mol Psychiatry.

[44] Sagiv SK, Kalkbrenner AE, Bellinger DC (2015). Of decrements and disorders: assessing impairments in neurodevelopment in prospective studies of environmental toxicant exposures. Environ Health.

[45] Robinson EB (2011). Evidence that autistic traits show the same etiology in the general population and at the quantitative extremes (5%, 2.5%, and 1%). Arch Gen Psychiatry.

[46] Lundström S (2012). Autism spectrum disorders and autisticlike traits: similar etiology in the extreme end and the normal variation. Arch Gen Psychiatry.

