# Supplementary material for: Meconium androgens are correlated with ASD-related phenotypic traits in early childhood in a familial enriched risk cohort
Source: Mol Autism. 2020 Nov 23;11:93. doi: 10.1186/s13229-020-00395-6 (PMC7686740; doi:10.1186/s13229-020-00395-6)
Supplement: Supplementary file 1 — Additional file 1. Supplementary tables and figures. [file 13229_2020_395_MOESM1_ESM.docx]

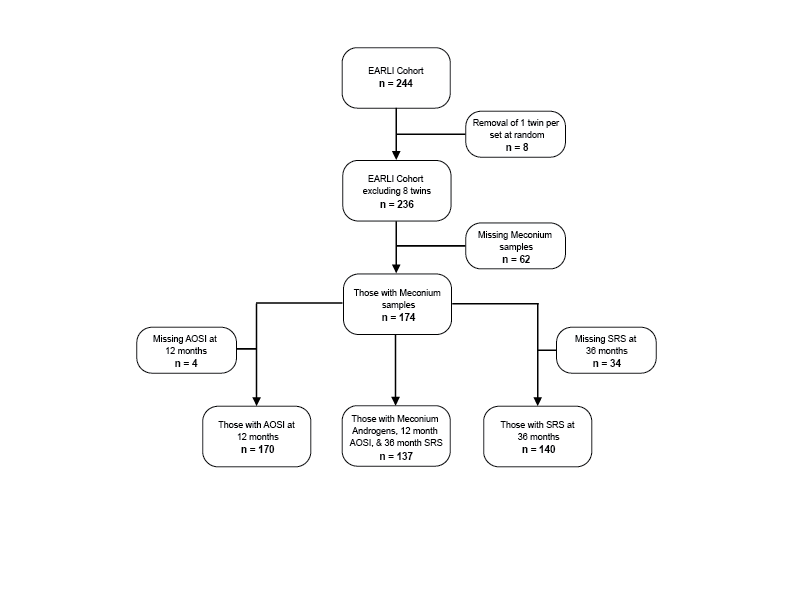
**Figure 1S**. Decision tree for inclusion and exclusion of participants. Availability of exposure and outcome measures led to a final sample of 170 participants for meconium and AOSI, and 140 for meconium and SRS.


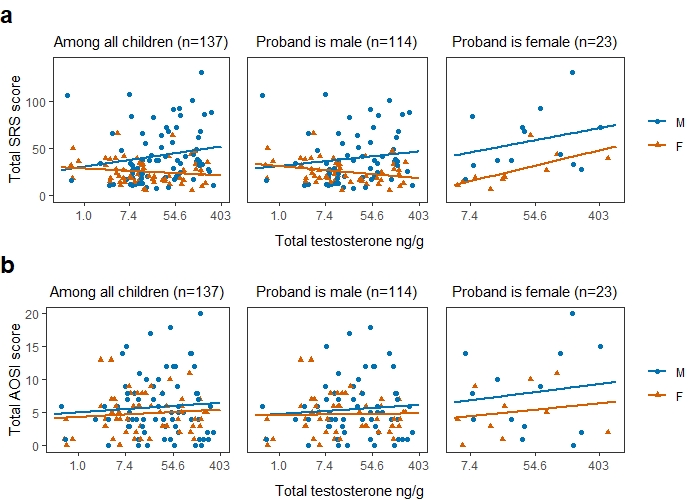


**Figure 2S**. Relationship between meconium total testosterone (T) and 12- and 36-month outcomes by sex, and further stratified by proband’s sex. **a** Total T levels vs score on the social responsiveness scale (SRS). **b** Autism Observation Scale for Infants (AOSI).


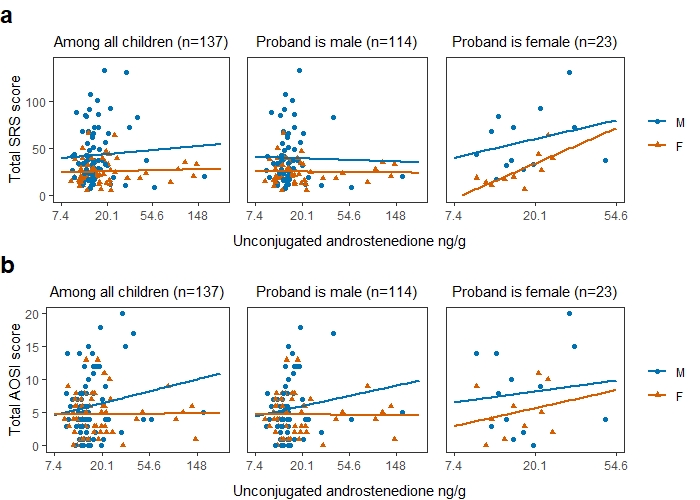


**Figure 3S**. Relationship between meconium unconjugated androstenedione (A4) and 12- and 36-month outcomes by sex, and further stratified by proband’s sex. **a** Unconjugated A4 levels vs score on the social responsiveness scale (SRS). **b** Autism Observation Scale for Infants (AOSI).


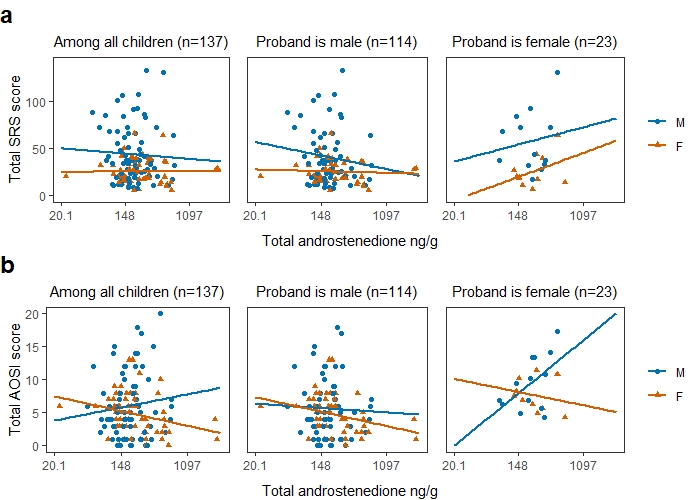


**Figure 4S**. Relationship between meconium total androstenedione (A4) and 12- and 36-month outcomes by sex, and further stratified by proband’s sex. **a** Total A4 levels vs score on the social responsiveness scale (SRS). **b** Autism Observation Scale for Infants (AOSI).


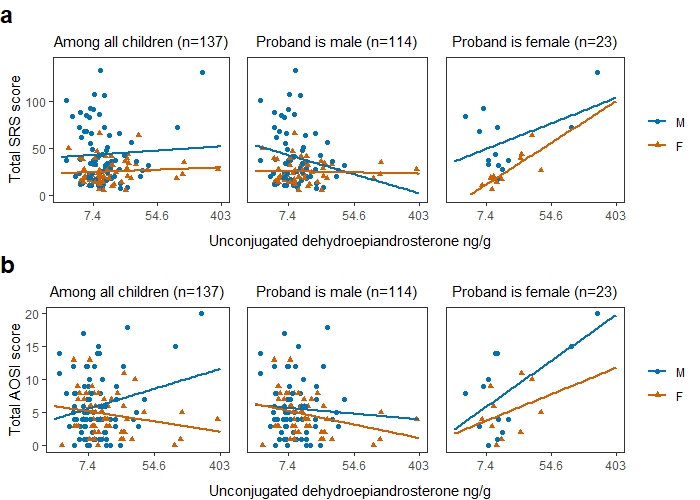


**Figure 5S**. Relationship between meconium unconjugated dehydroepiandrosterone (DHEA) and 12- and 36-month outcomes by sex, and further stratified by proband’s sex. **a** Unconjugated DHEA levels vs score on the social responsiveness scale (SRS). **b** Autism Observation Scale for Infants (AOSI).


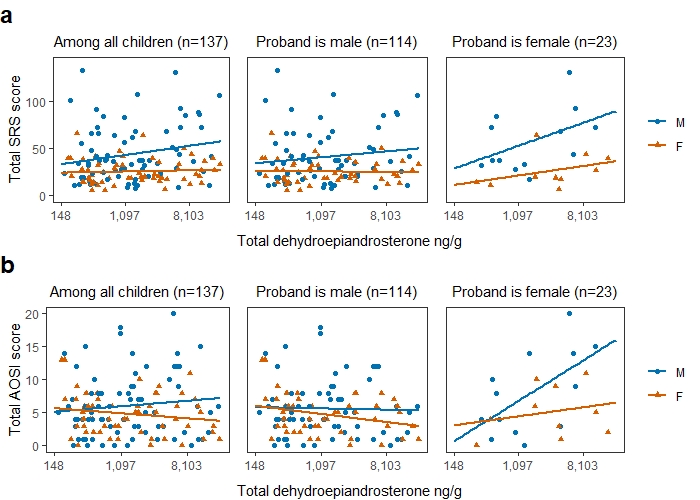


**Figure 6S**. Relationship between meconium total dehydroepiandrosterone (DHEA) and 12- and 36-month outcomes by sex, and further stratified by proband’s sex. **a** Total DHEA levels vs score on the social responsiveness scale (SRS). **b** Autism Observation Scale for Infants (AOSI).

Table 1S Unadjusted and adjusted log-log models of androgen levels and AOSI score at 12 months by sex of the child and sex of the proband.

| Hormones | Proband’s  sex | Subject’s sex | ln(AOSI+1) | | | | | | | | |
| --- | --- | --- | --- | --- | --- | --- | --- | --- | --- | --- | --- |
|  |  |  | Unadjusted model | | | | Adjusted^a^ model | | | | |
|  |  |  | Beta | 95% CI | p | R-square | Beta | 95% CI | p | R-square | *P*^b^ |
| ln(u-T) | All | Female (n=61) | 0.02 | -0.06, 0.11 | 0.62 | 0.00 | 0.02 | -0.06, 0.11 | 0.62 | 0.02 | 0.64 |
|  |  | Male (n=76) | 0.01 | -0.08, 0.11 | 0.79 | 0.00 | 0.01 | -0.08, 0.10 | 0.82 | 0.06 |  |
|  |  | All (n=137) | 0.03 | -0.04, 0.09 | 0.40 | 0.00 | 0.02 | -0.04, 0.08 | 0.57 | 0.04 |  |
|  | Male | Female (n=51) | 0.02 | -0.07, 0.11 | 0.64 | 0.00 | 0.03 | -0.06, 0.12 | 0.49 | 0.04 |  |
|  |  | Male (n=63) | 0.01 | -0.09, 0.11 | 0.82 | 0.00 | 0.01 | -0.09, 0.11 | 0.89 | 0.07 |  |
|  |  | All (n=114) | 0.03 | -0.04, 0.09 | 0.44 | 0.00 | 0.02 | -0.05, 0.09 | 0.56 | 0.04 |  |
|  | Female | Female (n=10) | 0.02 | -0.29, 0.34 | 0.89 | 0.00 | 0.23 | -0.12, 0.58 | 0.19 | 0.32 |  |
|  |  | Male (n=13) | 0.03 | -0.22, 0.27 | 0.82 | 0.00 | -0.03 | -0.34, 0.27 | 0.82 | 0.13 |  |
|  |  | All (n=23) | 0.03 | -0.16, 0.22 | 0.75 | 0.00 | 0.06 | -0.18, 0.29 | 0.63 | 0.11 |  |
| ln(u-A4) | All | Female (n=61) | 0.00 | -0.25, 0.26 | 0.98 | 0.00 | -0.05 | -0.31, 0.22 | 0.74 | 0.02 | 0.58 |
|  |  | Male (n=76) | 0.12 | -0.23, 0.48 | 0.51 | 0.01 | 0.10 | -0.26, 0.46 | 0.59 | 0.07 |  |
|  |  | All (n=137) | 0.05 | -0.17, 0.26 | 0.66 | 0.00 | 0.02 | -0.20, 0.24 | 0.85 | 0.04 |  |
|  | Male | Female (n=51) | 0.00 | -0.25, 0.25 | 0.98 | 0.00 | -0.04 | -0.30, 0.22 | 0.75 | 0.03 |  |
|  |  | Male (n=63) | 0.11 | -0.25, 0.47 | 0.54 | 0.01 | 0.04 | -0.34, 0.43 | 0.82 | 0.08 |  |
|  |  | All (n=114) | 0.04 | -0.17, 0.25 | 0.72 | 0.00 | 0.01 | -0.21, 0.23 | 0.94 | 0.04 |  |
|  | Female | Female (n=10) | 0.66 | -0.28, 2.49 | 0.48 | 0.05 | **2.02** | **0.47, 3.56** | **0.01** | **0.57** |  |
|  |  | Male (n=13) | 0.10 | -1.19, 1.40 | 0.87 | 0.00 | 0.06 | -1.37, 1.50 | 0.93 | 0.13 |  |
|  |  | All (n=23) | 0.26 | -0.77, 1.28 | 0.62 | 0.01 | 0.52 | -0.60, 1.63 | 0.36 | 0.14 |  |
| ln(u-DHEA) | All | Female (n=61) | -0.19 | -0.39, 0.01 | 0.07 | 0.04 | **-0.28** | **-0.49, -0.08** | **0.006** | **0.11** | 0.38 |
|  |  | Male (n=76) | 0.14 | -0.14, 0.42 | 0.31 | 0.01 | 0.21 | -0.05, 0.48 | 0.12 | 0.09 |  |
|  |  | All (n=137) | -0.08 | -0.26, 0.09 | 0.34 | 0.00 | -0.11 | -0.29, 0.06 | 0.21 | 0.05 |  |
|  | Male | Female (n=51) | **-0.26** | **-0.47, -0.06** | **0.01** | **0.08** | **-0.35** | **-0.55, -0.15** | **0.001** | **0.18** |  |
|  |  | Male (n=63) | -0.08 | -0.45, 0.29 | 0.68 | 0.00 | 0.00 | -0.37, 0.37 | 0.99 | 0.07 |  |
|  |  | All (n=114) | **-0.19** | **-0.37, -0.01** | **0.04** | **0.03** | **-0.24** | **-0.43, -0.05** | **0.01** | **0.08** |  |
|  | Female | Female (n=10) | 0.31 | -0.63, 1.25 | 0.51 | 0.06 | **1.09** | **0.22, 1.96** | **0.01** | **0.58** |  |
|  |  | Male (n=13) | 0.32 | -0.13, 0.78 | 0.16 | 0.17 | 0.35 | -0.17, 0.88 | 0.19 | 0.28 |  |
|  |  | All (n=23) | 0.33 | -0.07, 0.72 | 0.11 | 0.12 | **0.49** | **0.06, 0.92** | **0.02** | **0.30** |  |
| ln(t-T) | All | Female (n=61) | 0.02 | -0.09, 0.12 | 0.75 | 0.00 | 0.02 | -0.09, 0.12 | 0.79 | 0.02 | 0.90 |
|  |  | Male (n=76) | 0.04 | -0.08, 0.17 | 0.50 | 0.01 | 0.05 | -0.08, 0.17 | 0.48 | 0.07 |  |
|  |  | All (n=137) | 0.04 | -0.03, 0.13 | 0.28 | 0.01 | 0.03 | -0.05, 0.12 | 0.43 | 0.04 |  |
|  | Male | Female (n=51) | 0.01 | -0.10, 0.12 | 0.83 | 0.00 | 0.02 | -0.09, 0.14 | 0.72 | 0.03 |  |
|  |  | Male (n=63) | 0.04 | -0.09, 0.17 | 0.52 | 0.01 | 0.04 | -0.09, 0.17 | 0.57 | 0.08 |  |
|  |  | All (n=114) | 0.04 | -0.04, 0.12 | 0.36 | 0.01 | 0.03 | -0.06, 0.12 | 0.49 | 0.04 |  |
|  | Female | Female (n=10) | 0.09 | -0.34, 0.51 | 0.69 | 0.02 | **0.48** | **0.10, 0.87** | **0.014** | **0.52** |  |
|  |  | Male (n=13) | -0.01 | -0.44, 0.43 | 0.97 | 0.00 | -0.14 | -0.73, 0.45 | 0.64 | 0.15 |  |
|  |  | All (n=23) | 0.05 | -0.25, 0.35 | 0.75 | 0.00 | 0.19 | -0.20, 0.59 | 0.34 | 0.13 |  |
| ln(t-A4) | All | Female (n=61) | **-0.26** | **-0.46, -0.06** | **0.01** | **0.09** | **-0.31** | **-0.50, -0.11** | **0.002** | **0.13** | **0.01** |
|  |  | Male (n=76) | 0.07 | -0.30, 0.44 | 0.70 | 0.00 | 0.11 | -0.25, 0.47 | 0.54 | 0.07 |  |
|  |  | All (n=137) | -0.16 | -0.35,0.04 | 0.12 | 0.02 | -0.18 | -0.37, 0.02 | 0.08 | 0.06 |  |
|  | Male | Female (n=51) | **-0.25** | **-0.44, -0.06** | **0.01** | **0.10** | **-0.29** | **-0.47, -0.09** | **0.003** | **0.16** |  |
|  |  | Male (n=63) | -0.06 | -0.46, 0.33 | 0.75 | 0.00 | -0.03 | -0.42, 0.36 | 0.87 | 0.07 |  |
|  |  | All (n=114) | **-0.19** | **-0.38, 0.00** | **0.05** | **0.03** | **-0.21** | **-0.41, -0.02** | **0.03** | **0.08** |  |
|  | Female | Female (n=10) | -0.94 | -1.93, 0.05 | 0.06 | 0.18 | -0.58 | -2.15, 0.99 | 0.47 | 0.23 |  |
|  |  | Male (n=13) | 0.76 | -0.04, 1.57 | 0.06 | 0.20 | 0.67 | -0.36, 1.70 | 0.20 | 0.24 |  |
|  |  | All (n=23) | 0.31 | -0.46, 1.08 | 0.43 | 0.02 | 0.28 | -0.62, 1.18 | 0.54 | 0.12 |  |
| ln(t-DHEA) | All | Female (n=61) | -0.07 | -0.19, 0.05 | 0.25 | 0.02 | -0.10 | -0.22, 0.02 | 0.11 | 0.05 | 0.62 |
|  |  | Male (n=76) | 0.04 | -0.12, 0.19 | 0.66 | 0.00 | 0.00 | -0.15, 0.15 | 0.99 | 0.06 |  |
|  |  | All (n=137) | -0.03 | -0.12, 0.07 | 0.60 | 0.00 | -0.05 | -0.15, 0.04 | 0.27 | 0.05 |  |
|  | Male | Female (n=51) | -0.09 | -0.22, 0.04 | 0.18 | 0.03 | -0.08 | -0.24, 0.08 | 0.31 | 0.09 |  |
|  |  | Male (n=63) | -0.04 | -0.20, 0.12 | 0.63 | 0.00 | -0.06 | -0.20, 0.08 | 0.38 | 0.05 |  |
|  |  | All (n=114) | -0.07 | -0.17, 0.03 | 0.16 | 0.01 | **-0.11** | **-0.21, -0.01** | **0.037** | **0.07** |  |
|  | Female | Female (n=10) | 0.18 | -0.30, 0.65 | 0.47 | 0.06 | 0.35 | -0.06, 0.76 | 0.09 | 0.38 |  |
|  |  | Male (n=13) | 0.35 | -0.06, 0.76 | 0.09 | 0.22 | 0.36 | -0.11, 0.84 | 0.14 | 0.29 |  |
|  |  | All (n=23) | 0.23 | -0.09, 0.55 | 0.16 | 0.09 | **0.37** | **0.06, 0.68** | **0.018** | **0.28** |  |

^a^All models are adjusted for maternal and gestational age. In addition to maternal and gestational age the models collapsed on sex of the infant were adjusted for infant’s sex.

^b^P-value of the triple interaction term (androgen by child’s sex by proband’s sex) comes from the models that were fit in the entire sample (both subjects’ sexes, both probands’ sexes) and included covariates and the following interaction terms: 1) androgen by child’s sex, 2) androgen by proband’s sex, 3) child’s sex by proband’s sex, 4) androgen by child’s sex by proband’s sex.

Table 2S. Unadjusted and adjusted models of ln-transformed androgen levels and total AOSI score at 12 months by sex of the child and sex of the proband.

| Hormones | Proband’s  sex | Subject’s sex | Outcome=AOSI | | | | | | | | |
| --- | --- | --- | --- | --- | --- | --- | --- | --- | --- | --- | --- |
|  |  |  | Unadjusted model | | | | Adjusted^a^ model | | | | |
|  |  |  | Beta | 95% CI | p | R-square | Beta | 95% CI | p | R-square | *P*^b^ |
| ln(u-T) | All | Female (n=61) | 0.17 | -0.25, 0.59 | 0.42 | 0.01 | 0.16 | -0.26, 0.59 | 0.45 | 0.04 | 0.26 |
|  |  | Male (n=76) | 0.02 | -0.51, 0.55 | 0.95 | 0.00 | 0.03 | -0.49, 0.55 | 0.90 | 0.08 |  |
|  |  | All (n=137) | 0.07 | -0.25, 0.39 | 0.66 | 0.00 | 0.06 | -0.27, 0.39 | 0.74 | 0.03 |  |
|  | Male | Female (n=51) | 0.18 | -0.24, 0.60 | 0.39 | 0.01 | 0.22 | -0.20, 0.63 | 0.31 | 0.07 |  |
|  |  | Male (n=63) | 0.06 | -0.47, 0.59 | 0.83 | 0.00 | 0.07 | 0.46, 0.61 | 0.79 | 0.08 |  |
|  |  | All (n=114) | 0.11 | -0.21, 0.42 | 0.51 | 0.00 | 0.11 | -0.22, 0.44 | 0.52 | 0.03 |  |
|  | Female | Female (n=10) | 0.21 | -1,41, 1.82 | 0.80 | 0.01 | 0.88 | -0.96, 2.72 | 0.35 | 0.25 |  |
|  |  | Male (n=13) | 0.61 | -1.06, 2.28 | 0.47 | 0.05 | 0.23 | -1.85, 2.31 | 0.83 | 0.17 |  |
|  |  | All (n=23) | 0.40 | -0.83, 1.63 | 0.52 | 0.02 | 0.39 | -1.05, 1.83 | 0.60 | 0.14 |  |
| ln(u-A4) | All | Female (n=61) | 0.07 | -0.24, 1.38 | 0.92 | 0.00 | -0.11 | -1.44, 1.23 | 0.88 | 0.03 | 0.39 |
|  |  | Male (n=76) | 0.37 | -1.64, 2.37 | 0.72 | 0.00 | 0.63 | -1.43, 2.70 | 0.55 | 0.08 |  |
|  |  | All (n=137) | 0.11 | -0.98, 1.19 | 0.85 | 0.00 | 0.11 | -1.04, 1.26 | 0.85 | 0.03 |  |
|  | Male | Female (n=51) | 0.02 | -1.24, 1.28 | 0.97 | 0.00 | -0.10 | -1.36, 1.16 | 0.87 | 0.05 |  |
|  |  | Male (n=63) | 0.37 | -1.51, 2.26 | 0.70 | 0.00 | 0.45 | -1.68. 2.57 | 0.68 | 0.08 |  |
|  |  | All (n=114) | 0.12 | -0.91, 1.15 | 0.81 | 0.00 | 0.13 | -1.00, 1.25 | 0.83 | 0.03 |  |
|  | Female | Female (n=10) | 2.89 | -6.10, 11.88 | 0.53 | 0.05 | 7.00 | -2.66, 16.67 | 0.16 | 0.38 |  |
|  |  | Male (n=13) | 0.98 | -8.52, 10.48 | 0.84 | 0.00 | 0.15 | -10.03, 10.33 | 0.98 | 0.17 |  |
|  |  | All (n=23) | 1.26 | -5.40, 7.92 | 0.71 | 0.01 | 1.79 | -5.58, 9.17 | 0.63 | 0.13 |  |
| ln(u-DHEA) | All | Female (n=61) | -0.72 | -1.70, 0.26 | 0.15 | 0.03 | -1.01 | -2.04, 0.03 | 0.056 | 0.08 | 0.94 |
|  |  | Male (n=76) | -0.21 | -2.14, 1.73 | 0.83 | 0.00 | **2.17** | **0.53, 3.80** | **0.01** | **0.11** |  |
|  |  | All (n=137) | -0.76 | -1.66, 0.14 | 0.098 | 0.02 | -0.88 | -1.83, 0.07 | 0.07 | 0.05 |  |
|  | Male | Female (n=51) | -0.90 | -1.85, 0.04 | 0.06 | 0.06 | **-1.13** | **-2.15, 0.11** | **0.03** | **0.13** |  |
|  |  | Male (n=63) | -0.77 | -2.74, 1.20 | 0.44 | 0.01 | 0.46 | -1.62, 2.53 | 0.67 | 0.08 |  |
|  |  | All (n=114) | -0.82 | -1.70, 0.07 | 0.072 | 0.02 | **-0.99** | **-1.94, -0.04** | **0.042** | **0.05** |  |
|  | Female | Female (n=10) | 1.92 | -2.90, 6.73 | 0.44 | 0.08 | **5.06** | **0.23, 9.89** | **0.04** | **0.51** |  |
|  |  | Male (n=13) | **3.50** | **0.54, 6.45** | **0.02** | **0.33** | **3.62** | **0.34, 6.91** | **0.03** | **0.44** |  |
|  |  | All (n=23) | **3.32** | **0.85, 5.80** | **0.009** | **0.20** | **4.04** | **1.59, 6.48** | **0.001** | **0.41** |  |
| ln(t-T) | All | Female (n=61) | 0.21 | -0.30, 0.73 | 0.42 | 0.01 | 0.23 | -0.30, 0.76 | 0.40 | 0.04 | 0.13 |
|  |  | Male (n=76) | 0.24 | -0.47, 0.96 | 0.50 | 0.01 | 0.29 | -0.42, 0.99 | 0.43 | 0.08 |  |
|  |  | All (n=137) | 0.18 | -0.24, 0.59 | 0.40 | 0.00 | 0.17 | -0.26, 0.60 | 0.43 | 0.03 |  |
|  | Male | Female (n=51) | 0.22 | -0.30, 0.73 | 0.41 | 0.01 | 0.29 | -0.23, 0.81 | 0.27 | 0.07 |  |
|  |  | Male (n=63) | 0.26 | -0.42, 0.94 | 0.45 | 0.01 | 0.27 | -0.42, 0.97 | 0.44 | 0.09 |  |
|  |  | All (n=114) | 0.19 | -0.21, 0.59 | 0.35 | 0.01 | 0.21 | -0.22, 0.63 | 0.34 | 0.03 |  |
|  | Female | Female (n=10) | 0.47 | -1.61, 2.55 | 0.66 | 0.03 | 1.62 | -0.94, 4.18 | 0.22 | 0.33 |  |
|  |  | Male (n=13) | 0.57 | -2.44, 3.58 | 0.71 | 0.01 | -0.09 | -4.14, 3.96 | 0.97 | 0.17 |  |
|  |  | All (n=23) | 0.52 | -1.38, 2.42 | 0.59 | 0.01 | 0.50 | -1.97, 2.96 | 0.69 | 0.13 |  |
| ln(t-A4) | All | Female (n=61) | **-1.23** | **-2.21, -0.25** | **0.014** | **0.09** | **-1.37** | **-2.34, 0.40** | **0.006** | **0.14** | **0.007** |
|  |  | Male (n=76) | 0.09 | -2.02, 2.21 | 0.93 | 0.00 | 0.85 | -1.25, 2.95 | 0.43 | 0.08 |  |
|  |  | All (n=137) | -0.85 | -1.83, 0.12 | 0.086 | 0.02 | -0.91 | -1.91, 0.10 | 0.078 | 0.05 |  |
|  | Male | Female (n=51) | **-1.11** | **-2.06, -0.16** | **0.02** | **0.10** | **-1.19** | **-2.15, -0.24** | **0.01** | **0.15** |  |
|  |  | Male (n=63) | -0.46 | -2.51, 1.59 | 0.66 | 0.00 | 0.18 | -0.99, 2.34 | 0.87 | 0.08 |  |
|  |  | All (n=114) | -0.86 | -1.81, 0.08 | 0.073 | 0.03 | -0.96 | -1.95, 0.03 | 0.058 | 0.05 |  |
|  | Female | Female (n=10) | -2.15 | -7.67, 3.38 | 0.45 | 0.06 | -0.15 | -8.32, 8.02 | 0.97 | 0.15 |  |
|  |  | Male (n=13) | **6.69** | **0.17, 13.20** | **0.04** | **0.26** | 5.73 | -1.39, 12.84 | 0.11 | 0.35 |  |
|  |  | All (n=23) | 2.23 | -2.77, 7.23 | 0.38 | 0.03 | 3.09 | -2.44, 8.62 | 0.27 | 0.18 |  |
| ln(t-DHEA) | All | Female (n=61) | -0.27 | -0.89, 0.35 | 0.40 | 0.01 | -0.41 | -1.03, 0.21 | 0.20 | 0.05 | 0.26 |
|  |  | Male (n=76) | 0.29 | -0.59, 1.16 | 0.52 | 0.00 | 0.07 | -0.79, 0.94 | 0.87 | 0.08 |  |
|  |  | All (n=137) | -0.10 | -0.60, 0.40 | 0.70 | 0.00 | -0.20 | -0.72, 0/32 | 0.45 | 0.03 |  |
|  | Male | Female (n=51) | -0.37 | -1.03, 0.28 | 0.26 | 0.02 | -0.48 | -1.12, 0.15 | 0.14 | 0.09 |  |
|  |  | Male (n=63) | -0.01 | -0.84, 0.83 | 0.99 | 0.00 | -0.21 | -1.08, 0.65 | 0.63 | 0.08 |  |
|  |  | All (n=114) | -0.24 | -0.74, 0.26 | 0.34 | 0.01 | -0.32 | -0.84, 0.20 | 0.23 | 0.03 |  |
|  | Female | Female (n=10) | 0.66 | -1.68, 3.00 | 0.58 | 0.04 | 0.99 | -1.43, 3.42 | 0.42 | 0.24 |  |
|  |  | Male (n=13) | 3.02 | 0.09, 5.95 | 0.04 | 0.30 | 2.87 | -0.41, 6.14 | 0.09 | 0.37 |  |
|  |  | All (n=23) | 1.46 | -0.50, 3.42 | 0.14 | 0.09 | 1.85 | -0.29, 3.99 | 0.091 | 0.24 |  |

^a^All models are adjusted for maternal and gestational age. In addition to maternal and gestational age the models collapsed on sex of the infant were adjusted for infant’s sex.

^b^P- value of the robust Wald test ($R_{n}^{2}$ test) for the three-way interaction term (androgen by child’s sex by proband’s sex) comes from the models that were fit in the entire sample (both subjects’ sexes, both probands’ sexes) and included covariates and the following interaction terms: 1) androgen by child’s sex, 2) androgen by proband’s sex, 3) child’s sex by proband’s sex, 4) androgen by child’s sex by proband’s sex.

Table 3S. Unadjusted and adjusted log-log models of androgen levels and SRS score at 36 months by sex of the child and sex of the proband.

| Hormones | Proband’s  sex | Subject’s sex | Outcome = ln(SRS) | | | | | | | |  |
| --- | --- | --- | --- | --- | --- | --- | --- | --- | --- | --- | --- |
|  |  |  | Unadjusted model | | | | Adjusted^a^ model | | | |  |
|  |  |  | Beta | 95% CI | p | R-square | Beta | 95% CI | p | R-square | *P*^b^ |
| ln(u-T) | All | Female (n=61) | 0.01 | -0.07, 0.08 | 0.81 | 0.00 | 0.00 | -0.068, 0.08 | 0.96 | 0.01 | **0.01** |
|  |  | Male (n=76) | **0.11** | **0.02, 0.20** | **0.01** | **0.06** | **0.11** | **0.02, 0.20** | **0.015** | **0.10** |  |
|  |  | All (n=137) | **0.08** | **0.02, 0.14** | **0.006** | **0.04** | **0.08** | **0.02, 0.14** | **0.008** | **0.06** |  |
|  | Male | Female (n=51) | -0.03 | -0.11, 0.05 | 0.47 | 0.01 | -0.04 | -0.12, 0.03 | 0.28 | 0.05 |  |
|  |  | Male (n=63) | **0.12** | **0.02, 0.22** | **0.02** | 0.06 | **0.12** | **0.03, 0.20** | **0.009** | 0.12 |  |
|  |  | All (n=114) | 0.06 | -0.01, 0.12 | 0.08 | 0.02 | 0.03 | -0.03, 0.10 | 0.33 | 0.06 |  |
|  | Female | Female (n=10) | **0.26** | **0.15, 0.37** | **<.0001** | **0.55** | **0.25** | **0.06, 0.44** | **0.01** | **0.54** |  |
|  |  | Male (n=13) | 0.07 | -0.09, 0.22 | 0.40 | 0.06 | 0.04 | -0.12, 0.19 | 0.65 | 0.39 |  |
|  |  | All (n=23) | **0.16** | **0.01, 0.31** | **0.036** | **0.16** | 0.09 | -0.04, 0.22 | 0.17 | 0.54 |  |
| ln(u-A4) | All | Female (n=61) | 0.08 | -0.14, 0.30 | 0.49 | 0.01 | 0.06 | -0.17, 0.29 | 0.61 | 0.02 | 0.15 |
|  |  | Male (n=76) | 0.21 | -0.16, 0.58 | 0.26 | 0.01 | 0.25 | -0.12, 0.61 | 0.43 | 0.15 |  |
|  |  | All (n=137) | 0.05 | -0.17, 0.27 | 0.67 | 0.00 | 0.12 | -0.10, 0.33 | 0.28 | 0.10 |  |
|  | Male | Female (n=51) | 0.02 | -0.20, 0.24 | 0.83 | 0.00 | -0.00 | -0.23, 0.22 | 0.81 | 0.17 |  |
|  |  | Male (n=63) | 0.13 | -0.28, 0.54 | 0.53 | 0.00 | 0.21 | -0.21, 0.64 | 0.33 | 0.05 |  |
|  |  | All (n=114) | -0.02 | -0.25, 0.20 | 0.83 | 0.00 | 0.04 | -0.20, 0.27 | 0.77 | 0.06 |  |
|  | Female | Female (n=10) | **1.37** | **0.50, 2.24** | **0.002** | **0.46** | 1.16 | -0.12, 2.43 | 0.07 | 0.52 |  |
|  |  | Male (n=13) | 0.26 | -0.60, 1.12 | 0.56 | 0.04 | 0.22 | -0.52, 0.95 | 0.56 | 0.40 |  |
|  |  | All (n=23) | 0.75 | -0.04, 1.53 | 0.06 | 0.14 | 0.46 | -0.21, 1.14 | 0.18 | 0.52 |  |
| ln(u-DHEA) | All | Female (n=61) | 0.08 | -0.09, 0.25 | 0.35 | 0.02 | 0.07 | -0.12, 0.25 | 0.49 | 0.02 | 0.22 |
|  |  | Male (n=76) | 0.01 | -0.27, 0.29 | 0.95 | 0.00 | 0.04 | -0.25, 0.32 | 0.80 | 0.04 |  |
|  |  | All (n=137) | -0.00 | -0.17, 0.17 | 0.98 | 0.00 | 0.06 | -0.10, 0.22 | 0.45 | 0.10 |  |
|  | Male | Female (n=51) | 0.03 | -0.14, 0.19 | 0.76 | 0.00 | -0.03 | -0.22, 0.16 | 0.73 | 0.03 |  |
|  |  | Male (n=63) | -0.18 | -0.56, 0.19 | 0.34 | 0.02 | -0.13 | -0.51, 0.24 | 0.49 | 0.04 |  |
|  |  | All (n=114) | -0.07 | -0.24, 0.11 | 0.47 | 0.01 | -0.04 | -0.23, 0.15 | 0.67 | 0.06 |  |
|  | Female | Female (n=10) | **0.92** | **0.39, 1.45** | **0.001** | **0.54** | **0.85** | **0.24, 1.47** | **0.007** | **0.69** |  |
|  |  | Male (n=13) | 0.19 | -0.11, 0.48 | 0.21 | 0.12 | 0.15 | -0.15, 0.45 | 0.33 | 0.43 |  |
|  |  | All (n=23) | 0.33 | -0.01, 0.68 | 0.06 | 0.12 | 0.25 | -0.03, 0.54 | 0.08 | 0.54 |  |
| ln(t-T) | All | Female (n=61) | -0.03 | -0.13, 0.06 | 0.47 | 0.01 | -0.04 | -0.14, 0.05 | 0.37 | 0.02 | **0.03** |
|  |  | Male (n=76) | **0.16** | **0.04, 0.28** | **0.01** | **0.07** | **0.16** | **0.04, 0.28** | **0.009** | **0.11** |  |
|  |  | All (n=137) | **0.08** | **0.00, 0.16** | **0.05** | **0.03** | 0.05 | -0.03, 0.12 | 0.26 | 0.10 |  |
|  | Male | Female (n=51) | -0.09 | -0.18, 0.00 | 0.059 | 0.06 | **-0.11** | **-0.20, -0.01** | **0.02** | **0.11** |  |
|  |  | Male (n=63) | **0.15** | **0.001, 0.28** | **0.04** | **0.06** | **0.17** | **0.04, 0.30** | **0.01** | 0.11 |  |
|  |  | All (n=114) | 0.03 | -0.05, 0.12 | 0.45 | 0.00 | 0.00 | -0.08, 0.09 | 0.92 | 0.06 |  |
|  | Female | Female (n=10) | **0.31** | **0.10, 0.51** | **0.003** | **0.50** | 0.29 | -0.05, 0.64 | 0.10 | 0.53 |  |
|  |  | Male (n=13) | 0.13 | -0.15, 0.40 | 0.37 | 0.07 | 0.17 | -0.13, 0.47 | 0.26 | 0.44 |  |
|  |  | All (n=23) | **0.27** | **0.05, 0.49** | **0.014** | **0.22** | 0.20 | -0.02, 0.42 | 0.07 | 0.55 |  |
| ln(t-A4) | All | Female (n=61) | 0.01 | -0.19, 0.20 | 0.94 | 0.00 | -0.03 | -0.23, 0.18 | 0.80 | 0.01 | 0.80 |
|  |  | Male (n=76) | -0.17 | -0.53, 0.20 | 0.37 | 0.01 | -0.07 | -0.43, 0.29 | 0.70 | 0.04 |  |
|  |  | All (n=137) | -0.09 | -0.29, 0.12 | 0.41 | 0.01 | -0.05 | -0.25, 0.14 | 0.61 | 0.10 |  |
|  | Male | Female (n=51) | 0.00 | -0.19, 0.19 | 0.99 | 0.00 | -0.06 | -0.26, 0.15 | 0.58 | 0.03 |  |
|  |  | Male (n=63) | -0.23 | -0.64, 0.17 | 0.26 | 0.02 | -0.13 | -0.54, 0.28 | 0.34 | 0.18 |  |
|  |  | All (n=114) | -0.10 | -0.31, 0.11 | 0.34 | 0.01 | -0.10 | -0.30, 0.11 | 0.37 | 0.07 |  |
|  | Female | Female (n=10) | 0.30 | -0.68, 1.28 | 0.55 | 0.05 | -0.22 | -1.44, 0.99 | 0.72 | 0.35 |  |
|  |  | Male (n=13) | -0.02 | -0.76, 0.73 | 0.96 | 0.00 | -0.17 | -0.73, 0.40 | 0.57 | 0.40 |  |
|  |  | All (n=23) | 0.05 | -0.65, 0.76 | 0.88 | 0.00 | -0.12 | -0.66, 0.41 | 0.66 | 0.48 |  |
| ln(t-DHEA) | All | Female (n=61) | 0.05 | -0.06, 0.15 | 0.37 | 0.01 | 0.04 | -0.07, 0.15 | 0.45 | 0.02 | 0.43 |
|  |  | Male (n=76) | **0.16** | **0.02, 0.30** | **0.03** | **0.06** | **0.15** | **0.00, 0.29** | **0.048** | **0.09** |  |
|  |  | All (n=137) | 0.08 | -0.01, 0.18 | 0.08 | 0.02 | **0.09** | **0.00, 0.18** | **0.049** | **0.12** |  |
|  | Male | Female (n=51) | 0.01 | -0.10, 0.12 | 0.86 | 0.00 | 0.00 | -0.11, 0.11 | 0.999 | 0.02 |  |
|  |  | Male (n=63) | 0.13 | -0.03, 0.29 | 0.11 | 0.04 | 0.13 | -0.03, 0.29 | 0.12 | 0.07 |  |
|  |  | All (n=114) | 0.06 | -0.04, 0.17 | 0.22 | 0.01 | 0.07 | -0.04, 0.17 | 0.21 | 0.07 |  |
|  | Female | Female (n=10) | 0.26 | -0.03, 0.54 | 0.08 | 0.27 | 0.14 | -0.23, 0.52 | 0.45 | 0.38 |  |
|  |  | Male (n=13) | 0.21 | -0.07, 0.49 | 0.15 | 0.17 | 0.14 | -0.12, 0.39 | 0.29 | 0.45 |  |
|  |  | All (n=23) | 0.13 | -0.14, 0.41 | 0.35 | 0.04 | 0.14 | -0.06, 0.34 | 0.16 | 0.52 |  |

^a^All models are adjusted for maternal and gestational age. In addition to maternal and gestational age the models collapsed on sex of the infant were adjusted for infant’s sex.

^b^P-value of the triple interaction term (androgen by child’s sex by proband’s sex) comes from the models that were fit in the entire sample (both subjects’ sexes, both probands’ sexes) and included covariates and the following interaction terms: 1) androgen by child’s sex, 2) androgen by proband’s sex, 3) child’s sex by proband’s sex, 4) androgen by child’s sex by proband’s sex.

Table 4S. Unadjusted and adjusted models of ln-transformed androgen levels and total SRS score at 36 months by sex of the child and sex of the proband.

| Hormones | Proband’s  sex | Subject’s sex | Outcome=SRS | | | | | | | | |
| --- | --- | --- | --- | --- | --- | --- | --- | --- | --- | --- | --- |
|  |  |  | Unadjusted model | | | | Adjusted^a^ model | | | | |
|  |  |  | Beta | 95% CI | p | R-square | Beta | 95% CI | p | R-square | *P*^b^ |
| ln(u-T) | All | Female (n=61) | 0.34 | -1.32, 1.99 | 0.69 | 0.00 | 0.17 | -1.53, 1.87 | 0.80 | 0.01 | 0.08 |
|  |  | Male (n=76) | **3.16** | **-0.05, 6.37** | **0.05** | **0.03** | **3.11** | **0.09, 6.13** | **0.04** | **0.09** |  |
|  |  | All (n=137) | **1.67** | **0.17, 3.17** | **0.03** | **0.02** | 1.31 | -0.29, 2.92 | 0.10 | 0.07 |  |
|  | Male | Female (n=51) | -0.60 | -2.35, 1.17 | 0.51 | 0.01 | -0.79 | -2.57, 0.99 | 0.38 | 0.04 |  |
|  |  | Male (n=63) | 3.27 | -0.08, 6.63 | 0.06 | 0.04 | **3.66** | **0.59, 6.73** | **0.02** | **0.11** |  |
|  |  | All (n=114) | 0.79 | -0.72, 2.30 | 0.30 | 0.01 | 0.70 | -0.97, 2.37 | 0.41 | 0.03 |  |
|  | Female | Female (n=10) | **5.48** | **3.34, 7.62** | **<0.0001** | **0.52** | **5.76** | **2.23, 9.30** | **0.001** | **0.46** |  |
|  |  | Male (n=13) | 2.76 | -5.8, 11.33 | 0.53 | 0.03 | 0.02 | -7.86, 7.90 | 0.99 | 0.35 |  |
|  |  | All (n=23) | 4.20 | -1.15, 9.55 | 0.12 | 0.07 | 0.26 | -5.08, 5.60 | 0.92 | 0.41 |  |
| ln(u-A4) | All | Female (n=61) | 1.83 | -3.12, 6.78 | 0.47 | 0.01 | 1.33 | -3.85, 6.52 | 0.61 | 0.02 | 0.17 |
|  |  | Male (n=76) | 9.24 | -4.41, 22.89 | 0.18 | 0.01 | 9.70 | -3.95, 23.35 | 0.61 | 0.06 |  |
|  |  | All (n=137) | -0.82 | -6.34, 4.71 | 0.77 | 0.00 | 0.38 | -5.64, 6.40 | 0.90 | 0.06 |  |
|  | Male | Female (n=51) | 0.78 | -4.24, 5.79 | 0.76 | 0.00 | 0.12 | -5.08, 5.32 | 0.96 | 0.02 |  |
|  |  | Male (n=63) | 4.70 | -9.52, 18.91 | 0.52 | 0.00 | 5.51 | -9.40,20.42 | 0.47 | 0.05 |  |
|  |  | All (n=114) | -1.90 | -7.08, 3.28 | 0.47 | 0.00 | -1.55 | -7.40, 4.29 | 0.60 | 0.03 |  |
|  | Female | Female (n=10) | **33.62** | **6.56, 60.68** | **0.01** | **0.37** | 29.71 | -6.88, 66.31 | 0.11 | 0.46 |  |
|  |  | Male (n=13) | 13.15 | -32.87, 59.17 | 0.58 | 0.03 | 7.96 | -29.50, 45.42 | 0.68 | 0.36 |  |
|  |  | All (n=23) | 20.06 | -10.89, 51.00 | 0.20 | 0.06 | 9.37 | -16.32, 35.05 | 0.47 | 0.41 |  |
| ln(u-DHEA) | All | Female (n=61) | 1.70 | -2.03, 5.44 | 0.37 | 0.01 | 1.20 | -3.05, 5.45 | 0.58 | 0.02 | 0.001 |
|  |  | Male (n=76) | -6.28 | -17.51, 4.96 | 0.27 | 0.01 | -2.88 | -13.60, 7.83 | 0.60 | 0.05 |  |
|  |  | All (n=137) | 0.12 | -4.08, 4.33 | 0.95 | 0.00 | 0.93 | -3.55, 5.41 | 0.68 | 0.06 |  |
|  | Male | Female (n=51) | 0.68 | -3.16, 4.51 | 0.73 | 0.00 | -0.79 | -5.25, 3.68 | 0.73 | 0.03 |  |
|  |  | Male (n=63) | -6.78 | -18.77, 5.21 | 0.27 | 0.02 | -2.69 | -14.50, 9.11 | 0.65 | 0.05 |  |
|  |  | All (n=114) | -0.68 | -4.71, 3.35 | 0.74 | 0.00 | -0.66 | -5.23, 3.90 | 0.78 | 0.03 |  |
|  | Female | Female (n=10) | **33.45** | **23.70,43.20** | **<0.0001** | **0.47** | **41.95** | **29.76, 54.13** | **<0.0001** | **0.53** |  |
|  |  | Male (n=13) | 13.75 | -3.12, 30.63 | 0.11 | 0.16 | -7.24 | -25.50, 11.01 | 0.44 | 0.35 |  |
|  |  | All (n=23) | **21.14** | **8.71, 33.58** | **0.0009** | **0.1** | **13.95** | **1.37, 26.53** | **0.03** | **0.48** |  |
| ln(t-T) | All | Female (n=61) | -0.85 | -2.94, 1.24 | 0.43 | 0.01 | -1.06 | -3.18, 1.07 | 0.33 | 0.03 | 0.011 |
|  |  | Male (n=76) | **4.95** | **0.50, 9.41** | **0.03** | **0.04** | **4.65** | **0.49, 8.81** | **0.03** | **0.1** |  |
|  |  | All (n=137) | 1.43 | -0.57, 3.44 | 0.16 | 0.01 | 1.61 | -0.45, 3.67 | 0.13 | 0.03 |  |
|  | Male | Female (n=51) | -2.09 | -4.19, 0.03 | 0.051 | 0.06 | -2.43 | -4.56, -0.29 | 0.26 | 0.10 |  |
|  |  | Male (n=63) | 4.37 | -0.15, 8.9 | 0.058 | 0.04 | **4.41** | **0.24, 8.58** | **0.04** | **0.10** |  |
|  |  | All (n=114) | 0.08 | -1.87, 2.03 | 0.94 | 0.00 | 0.004 | -2.15, 2.16 | 1.00 | 0.03 |  |
|  | Female | Female (n=10) | **6.90** | **3.90, 9.90** | **<0.0001** | **0.49** | 7.3 | -0.11, 14.71 | 0.053 | 0.45 |  |
|  |  | Male (n=13) | 5.11 | -9.68, 19.90 | 0.50 | 0.04 | 4.88 | -9.80, 19.55 | 0.51 | 0.37 |  |
|  |  | All (n=23) | **7.39** | **-0.34, 15.12** | **0.06** | **0.12** | 3.31 | -5.16, 11.77 | 0.44 | 0.42 |  |
| ln(t-A4) | All | Female (n=61) | -0.11 | -4.44, 4.21 | 0.96 | 0.00 | -0.80 | -5.34, 3.74 | 0.73 | 0.01 | 0.25 |
|  |  | Male (n=76) | -9.70 | -22.17, 2.77 | 0.13 | 0.02 | -5.43 | -18.01, 7.14 | 0.40 | 0.06 |  |
|  |  | All (n=137) | -1.92 | -6.80, 2.96 | 0.44 | 0.00 | -1.51 | -6.68, 3.66 | 0.57 | 0.06 |  |
|  | Male | Female (n=51) | -0.05 | -4.38, 4.28 | 0.98 | 0.00 | -1.27 | -5.88, 3.34 | 0.59 | 0.03 |  |
|  |  | Male (n=63) | -9.67 | -22.63, 3.29 | 0.14 | 0.02 | -4.41 | -17.52, 8.69 | 0.51 | 0.05 |  |
|  |  | All (n=114) | -1.65 | -6.30, 3.01 | 0.49 | 0.00 | -1.90 | -7.01, 3.21 | 0.47 | 0.03 |  |
|  | Female | Female (n=10) | 10.91 | -14.78, 36.61 | 0.41 | 0.07 | -1.72 | -34.49, 31.06 | 0.92 | 0.28 |  |
|  |  | Male (n=13) | -2.34 | -41.64, 36.95 | 0.91 | 0.00 | -16.35 | -36.99. 4.28 | 0.12 | 0.43 |  |
|  |  | All (n=23) | -2.64 | -27.53, 22.25 | 0.84 | 0.00 | -9.24 | -27.11, 8.64 | 0.31 | 0.42 |  |
| ln(t-DHEA) | All | Female (n=61) | 1.32 | -1.02, 3.66 | 0.27 | 0.02 | 1.14 | -1.28, 3.57 | 0.36 | 0.03 | 0.93 |
|  |  | Male (n=76) | **6.15** | **0.99, 11.30** | **0.02** | **0.05** | **5.23** | **0.08, 10.39** | **0.05** | **0.08** |  |
|  |  | All (n=137) | 1.56 | -0.83, 3.95 | 0.20 | 0.01 | 1.78 | -0.77, 4.33 | 0.17 | 0.06 |  |
|  | Male | Female (n=51) | 0.57 | -2.02, 3.15 | 0.67 | 0.00 | 0.31 | -2.32, 2.94 | 0.82 | 0.02 |  |
|  |  | Male (n=63) | 4.94 | -0.41, 10.28 | 0.07 | 0.03 | 4.29 | -0.98, 9.56 | 0.11 | 0.07 |  |
|  |  | All (n = 114) | 0.84 | -1.54, 3.21 | 0.49 | 0.00 | 0.90 | -1.68, 3.48 | 0.49 | 0.03 |  |
|  | Female | Female (n=10) | **6.07** | **0.09, 12.04** | **0.047** | **0.25** | 3.36 | -6.15, 12.88 | 0.49 | 0.31 |  |
|  |  | Male (n=13) | 10.81 | -4.64, 26.27 | 0.17 | 0.15 | 6.08 | -7.95, 20.11 | 0.40 | 0.39 |  |
|  |  | All (n = 23) | 3.43 | -6.49, 13.35 | 0.50 | 0.02 | 2.68 | -5.30, 10.66 | 0.51 | 0.41 |  |

^a^All models are adjusted for maternal and gestational age. In addition to maternal and gestational age the models collapsed on sex of the infant were adjusted for infant’s sex

^b^P-value of the robust Wald test ($R_{n}^{2}$ test) for the three-way interaction term (androgen by child’s sex by proband’s sex) comes from the models that were fit in the entire sample (both subjects’ sexes, both probands’ sexes) and included covariates and the following interaction terms: 1) androgen by child’s sex, 2) androgen by proband’s sex, 3) child’s sex by proband’s sex, 4) androgen by child’s sex by proband’s sex.

Table S5. Unadjusted and adjusted log-log models of androgen levels and AOSI score at 12 months by sex of the child and sex of the proband with all available data: N=170.

| Hormones | Proband’s  sex | Subject’s sex | ln(AOSI+1) | | | | | | | |
| --- | --- | --- | --- | --- | --- | --- | --- | --- | --- | --- |
|  |  |  | Unadjusted model | | | | Adjusted^a^ model | | | |
|  |  |  | Beta | 95% CI | p | R-square | Beta | 95% CI | p | R-square |
| ln(u-T) | All | Female (n=74) | 0.05 | -0.03, 0.14 | 0.21 | 0.02 | 0.05 | -0.03, 0.14 | 0.24 | 0.03 |
|  |  | Male (n=96) | 0.02 | -0.06, 0.10 | 0.68 | 0.00 | 0.01 | -0.07, 0.09 | 0.72 | 0.04 |
|  |  | All (n=170) | 0.04 | -0.02, 0.09 | 0.21 | 0.01 | 0.03 | -0.03, 0.09 | 0.30 | 0.04 |
|  | Male | Female (n=62) | 0.05 | -0.03, 0.14 | 0.22 | 0.02 | 0.06 | -0.03, 0.15 | 0.17 | 0.04 |
|  |  | Male (n=80) | 0.02 | -0.07, 0.11 | 0.68 | 0.00 | 0.01 | -0.07, 0.10 | 0.77 | 0.05 |
|  |  | All (n=142) | 0.04 | -0.02, 0.10 | 0.22 | 0.01 | 0.03 | -0.03, 0.09 | 0.29 | 0.03 |
|  | Female | Female (n=12) | 0.08 | -0.25, 0.42 | 0.62 | 0.03 | 0.23 | -0.09, 0.56 | 0.16 | 0.41 |
|  |  | Male (n=16) | 0.03 | -0.16, 0.23 | 0.73 | 0.01 | -0.04 | -0.31, 0.23 | 0.79 | 0.07 |
|  |  | All (n=28) | 0.04 | -0.14, 0.22 | 0.65 | 0.01 | 0.08 | -0.13, 0.30 | 0.45 | 0.15 |
| ln(u-A4) | All | Female (n=74) | -0.00 | -0.27, 0.26 | 0.98 | 0.00 | -0.04 | -0.32, 0.23 | 0.76 | 0.02 |
|  |  | Male (n=96) | 0.17 | -0.14, 0.48 | 0.28 | 0.02 | 0.17 | -0.15, 0.48 | 0.30 | 0.05 |
|  |  | All (n=170) | 0.08 | -0.12, 0.28 | 0.44 | 0.00 | 0.06 | -0.15, 0.27 | 0.57 | 0.03 |
|  | Male | Female (n=62) | -0.01 | -0.26, 0.24 | 0.93 | 0.00 | -0.04 | -0.31, 0.22 | 0.74 | 0.02 |
|  |  | Male (n=80) | 0.18 | -0.13, 0.50 | 0.26 | 0.02 | 0.16 | -0.17, 0.50 | 0.34 | 0.06 |
|  |  | All (n=142) | 0.08 | -0.12, 0.28 | 0.43 | 0.00 | 0.06 | -0.15, 0.27 | 0.57 | 0.03 |
|  | Female | Female (n=12) | 0.72 | -0.21, 2.65 | 0.57 | 0.05 | **2.00** | **0.43, 3.57** | **0.02** | **0.59** |
|  |  | Male (n=16) | 0.04 | -0.02, 1.09 | 0.95 | 0.00 | -0.08 | -1.30, 1.14 | 0.90 | 0.06 |
|  |  | All (n=28) | 0.15 | -0.84, 1.14 | 0.76 | 0.00 | 0.39 | -0.68, 1.46 | 0.48 | 0.16 |
| ln(u-DHEA) | All | Female (n=74) | -0.12 | -0.32, 0.07 | 0.22 | 0.02 | -0.21 | -0.41, -0.00 | **0.05** | 0.05 |
|  |  | Male (n=96) | 0.16 | -0.08, 0.40 | 0.18 | 0.02 | 0.22 | -0.02, 0.45 | 0.07 | 0.06 |
|  |  | All (n=170) | -0.02 | -0.18, 0.14 | 0.78 | 0.00 | -0.03 | -0.19, 0.13 | 0.70 | 0.03 |
|  | Male | Female (n=62) | -0.15 | -0.34, 0.04 | 0.12 | 0.03 | **-0.23** | **-0.43, -0.03** | **0.03** | **0.07** |
|  |  | Male (n=80) | 0.05 | -0.26, 0.35 | 0.75 | 0.00 | 0.12 | -0.18, 0.41 | 0.44 | 0.05 |
|  |  | All (n=142) | -0.08 | -0.24, 0.09 | 0.36 | 0.00 | -0.11 | -0.28, 0.06 | 0.22 | 0.03 |
|  | Female | Female (n=12) | 0.18 | -0.85, 1.22 | 0.73 | 0.01 | **0.87** | **-0.06, 1,79** | **0.07** | **0.50** |
|  |  | Male (n=16) | 0.26 | -0.12, 0.63 | 0.18 | 0.12 | 0.26 | -0.18, 0.71 | 0.25 | 0.16 |
|  |  | All (n=28) | 0.23 | -0.16, 0.62 | 0.25 | 0.05 | 0.35 | -0.07, 0.76 | 0.10 | 0.22 |
| ln(t-T) | All | Female (n=74) | 0.05 | -0.05, 0.16 | 0.30 | 0.01 | 0.05 | -0.05, 0.16 | 0.33 | 0.02 |
|  |  | Male (n=96) | 0.02 | -0.09, 0.13 | 0.69 | 0.00 | 0.03 | -0.08, 0.13 | 0.65 | 0.04 |
|  |  | All (n=170) | 0.04 | -0.03, 0.12 | 0.28 | 0.01 | 0.04 | -0.04, 0.11 | 0.33 | 0.03 |
|  | Male | Female (n=62) | 0.05 | -0.05, 0.15 | 0.35 | 0.01 | 0.06 | -0.05, 0.16 | 0.30 | 0.03 |
|  |  | Male (n=80) | 0.02 | -0.09, 0.13 | 0.73 | 0.00 | 0.02 | -0.10, 0.13 | 0.78 | 0.05 |
|  |  | All (n=142) | 0.04 | -0.04, 0.11 | 0.33 | 0.01 | 0.03 | -0.04, 0.11 | 0.38 | 0.03 |
|  | Female | Female (n=12) | 0.13 | -0.32, 0.58 | 0.57 | 0.04 | 0.31 | -0.11, 0.74 | 0.15 | 0.43 |
|  |  | Male (n=16) | 0.01 | -0.32, 0.34 | 0.95 | 0.00 | -0.18 | -0.69, 0.33 | 0.48 | 0.09 |
|  |  | All (n=28) | 0.05 | -0.22, 0.32 | 0.72 | 0.00 | 0.23 | -0.15, 0.58 | 0.24 | 0.16 |
| ln(t-A4) | All | Female (n=74) | **-0.25** | **-0.44, -0.06** | **0.01** | **0.07** | **-0.29** | **-0.48, -0.09** | **0.003** | **0.10** |
|  |  | Male (n=96) | 0.09 | -0.24, 0.43 | 0.58 | 0.00 | 0.11 | -0.22, 0.44 | 0.51 | 0.04 |
|  |  | All (n=170) | -0.14 | -0.32,0.04 | 0.13 | 0.01 | -0.16 | -0.34, 0.02 | 0.09 | 0.04 |
|  | Male | Female (n=62) | **-0.24** | **-0.41, -0.06** | **0.01** | **0.08** | **-0.27** | **-0.44, -0.09** | **0.003** | **0.12** |
|  |  | Male (n=80) | -0.04 | -0.40, 0.31 | 0.82 | 0.00 | -0.03 | -0.38, 0.33 | 0.89 | 0.05 |
|  |  | All (n=142) | -0.17 | -0.35, 0.01 | 0.06 | 0.02 | **-0.19** | **-0.37, -0.01** | **0.04** | **0.05** |
|  | Female | Female (n=12) | **-1.19** | **-1.04, -0.34** | **0.01** | **0.27** | -0.69 | -1.88, 0.50 | 0.26 | 0.35 |
|  |  | Male (n=16) | **0.84** | **0.30, 1.37** | **0.002** | **0.27** | **0.79** | **0.03, 1.56** | **0.04** | **0.24** |
|  |  | All (n=28) | 0.22 | -0.54, 0.98 | 0.57 | 0.01 | 0.18 | -0.63, 1.00 | 0.66 | 0.14 |
| ln(t-DHEA) | All | Female (n=74) | -0.05 | -0.17, 0.07 | 0.39 | 0.01 | -0.08 | -0.20, 0.04 | 0.19 | 0.03 |
|  |  | Male (n=96) | 0.02 | -0.11, 0.15 | 0.76 | 0.00 | 0.01 | -0.13, 0.14 | 0.94 | 0.04 |
|  |  | All (n=170) | -0.02 | -0.11, 0.07 | 0.71 | 0.00 | -0.04 | -0.13, 0.05 | 0.42 | 0.03 |
|  | Male | Female (n=62) | -0.06 | -0.18, 0.07 | 0.36 | 0.01 | -0.08 | -0.21, 0.04 | 0.19 | 0.04 |
|  |  | Male (n=80) | -0.04 | -0.18, 0.10 | 0.57 | 0.00 | -0.06 | -0.20, 0.08 | 0.38 | 0.05 |
|  |  | All (n=142) | -0.05 | -0.14, 0.04 | 0.28 | 0.01 | -0.07 | -0.17, 0.02 | 0.13 | 0.04 |
|  | Female | Female (n=12) | 0.09 | -0.37, 0.54 | 0.72 | 0.01 | 0.11 | -0.30, 0.51 | 0.60 | 0.30 |
|  |  | Male (n=16) | 0.32 | -0.03, 0.66 | 0.07 | 0.19 | 0.36 | -0.06, 0.77 | 0.10 | 0.22 |
|  |  | All (n=28) | 0.19 | -0.11, 0.49 | 0.22 | 0.05 | 0.26 | -0.04, 0.56 | 0.08 | 0.22 |

^a^All models are adjusted for maternal and gestational age. In addition to maternal and gestational age the models collapsed on sex of the infant were adjusted for infant’s sex.

Table S6. Unadjusted and adjusted models of ln-transformed androgen levels and untransformed AOSI score at 12 months by sex of the child and sex of the proband with all available data: N=170.

| Hormones | Proband’s  sex | Subject’s sex | AOSI | | | | | | | |
| --- | --- | --- | --- | --- | --- | --- | --- | --- | --- | --- |
|  |  |  | Unadjusted model | | | | Adjusted^a^ model | | | |
|  |  |  | Beta | 95% CI | p | R-square | Beta | 95% CI | p | R-square |
| ln(u-T) | All | Female (n=74) | 0.27 | -0.12, 0.66 | 0.18 | 0.02 | 0.25 | -0.14, 0.65 | 0.21 | 0.04 |
|  |  | Male (n=96) | 0.03 | -0.42, 0.47 | 0.90 | 0.00 | 0.05 | -0.40, 0.49 | 0.84 | 0.04 |
|  |  | All (n=170) | 0.13 | -0.15, 0.41 | 0.37 | 0.00 | 0.11 | -0.18, 0.41 | 0.45 | 0.02 |
|  | Male | Female (n=62) | 0.28 | -0.12, 0.68 | 0.17 | 0.03 | 0.30 | -0.09, 0.70 | 0.13 | 0.07 |
|  |  | Male (n=80) | 0.10 | -0.34, 0.54 | 0.66 | 0.00 | 0.09 | -0.37, 0.56 | 0.69 | 0.04 |
|  |  | All (n=142) | 0.17 | -0.12, 0.45 | 0.25 | 0.01 | 0.17 | -0.13, 0.46 | 0.26 | 0.02 |
|  | Female | Female (n=12) | 0.36 | -1.15, 1.87 | 0.64 | 0.02 | 0.97 | -0.44, 2.39 | 0.18 | 0.38 |
|  |  | Male (n=16) | 0.54 | -0.88, 1.96 | 0.46 | 0.04 | 0.14 | -1.73, 2.01 | 0.88 | 0.10 |
|  |  | All (n=28) | 0.39 | -0.73, 1.50 | 0.49 | 0.01 | 0.46 | -0.88, 1.81 | 0.50 | 0.13 |
| ln(u-A4) | All | Female (n=74) | 0.05 | -1.24, 1.33 | 0.94 | 0.00 | -0.12 | -1.42, 1.19 | 0.86 | 0.02 |
|  |  | Male (n=96) | 0.71 | -1.00, 2.42 | 0.42 | 0.01 | 1.05 | -0.72, 2.82 | 0.24 | 0.05 |
|  |  | All (n=170) | 0.30 | -0.70, 1.31 | 0.55 | 0.00 | 0.31 | -0.75, 1.36 | 0.57 | 0.02 |
|  | Male | Female (n=62) | -0.01 | -1.27, 1.24 | 0.98 | 0.00 | -0.10 | -1.37, 1.16 | 0.87 | 0.03 |
|  |  | Male (n=80) | 0.85 | -0.75, 2.46 | 0.30 | 0.01 | 1.13 | -0.67, 2.92 | 0.22 | 0.05 |
|  |  | All (n=142) | 0.37 | -0.59, 1.33 | 0.45 | 0.00 | 0.38 | -0.65, 1.41 | 0.47 | 0.02 |
|  | Female | Female (n=12) | 2.50 | -6.51, 11.52 | 0.57 | 0.03 | 6.94 | -1.74, 15.61 | 0.12 | 0.44 |
|  |  | Male (n=16) | 1.04 | -6.37, 8.45 | 0.78 | 0.01 | -0.94 | -9.86, 7.97 | 0.84 | 0.09 |
|  |  | All (n=28) | 0.12 | -5.94, 6.18 | 0.97 | 0.00 | 0.77 | -6.05, 7.58 | 0.83 | 0.11 |
| ln(u-DHEA) | All | Female (n=74) | -0.42 | -1.36, 0.51 | 0.38 | 0.01 | -0.64 | -1.60, 0.32 | 0.19 | 0.04 |
|  |  | Male (n=96) | 0.11 | -1.46, 1.69 | 0.89 | 0.00 | **1.94** | **0.51, 3.37** | **0.008** | **0.06** |
|  |  | All (n=170) | -0.43 | -1.23, 0.36 | 0.29 | 0.01 | -0.45 | -1.27, 0.39 | 0.29 | 0.02 |
|  | Male | Female (n=62) | -0.48 | -1.40, 0.45 | 0.31 | 0.02 | -0.61 | -1.59, 0.38 | 0.23 | 0.05 |
|  |  | Male (n=80) | 0.04 | -1.52, 1.61 | 0.96 | 0.00 | 1.02 | -0.65, 2.69 | 0.23 | 0.05 |
|  |  | All (n=142) | -0.33 | -1.11, 0.46 | 0.42 | 0.00 | -0.40 | -1.23, 0.44 | 0.35 | 0.02 |
|  | Female | Female (n=12) | 1.16 | -3.52, 5.85 | 0.63 | 0.03 | **4.22** | **0.10, 8.34** | **0.04** | **0.51** |
|  |  | Male (n=16) | **3.10** | **0.65, 5.56** | **0.01** | **0.30** | 2.97 | -0.08, 6.02 | 0.06 | 0.30 |
|  |  | All (n=28) | **2.51** | **0.13, 4.90** | **0.04** | **0.10** | **3.23** | **0.87, 5.60** | **0.007** | **0.30** |
| ln(t-T) | All | Female (n=74) | 0.32 | -0.19, 0.82 | 0.22 | 0.02 | 0.31 | -0.20, 0.82 | 0.23 | 0.04 |
|  |  | Male (n=96) | 0.10 | -0.49, 0.70 | 0.74 | 0.00 | 0.16 | -0.44, 0.76 | 0.60 | 0.04 |
|  |  | All (n=170) | 0.17 | -0.21, 0.54 | 0.38 | 0.00 | 0.17 | -0.22, 0.55 | 0.39 | 0.02 |
|  | Male | Female (n=62) | 0.33 | -0.18, 0.83 | 0.20 | 0.03 | 0.39 | -0.12, 0.90 | 0.13 | 0.07 |
|  |  | Male (n=80) | 0.14 | -0.43, 0.71 | 0.63 | 0.00 | 0.16 | -0.45, 0.76 | 0.61 | 0.04 |
|  |  | All (n=142) | 0.19 | -0.18, 0.56 | 0.31 | 0.01 | 0.21 | -0.18, 0.59 | 0.29 | 0.02 |
|  | Female | Female (n=12) | 0.56 | -1.50, 2.63 | 0.59 | 0.03 | 1.20 | -0.82, 3.21 | 0.24 | 0.37 |
|  |  | Male (n=16) | 0.46 | -2.00, 2.92 | 0.72 | 0.01 | -0.45 | -3.97, 3.06 | 0.80 | 0.10 |
|  |  | All (n=28) | 0.45 | -1.22, 2.13 | 0.60 | 0.01 | 0.68 | -1.51, 2.86 | 0.54 | 0.12 |
| ln(t-A4) | All | Female (n=74) | **-1.21** | **-2.17, -0.26** | **0.01** | **0.08** | **-1.37** | **-2.30, -0.43** | **0.004** | **0.12** |
|  |  | Male (n=96) | 0.22 | -1.64, 2.09 | 0.82 | 0.00 | 0.64 | -1.26, 2.55 | 0.51 | 0.04 |
|  |  | All (n=170) | -0.80 | -1.69, 0.10 | 0.08 | 0.02 | -0.89 | -1.80, 0.02 | 0.06 | 0.04 |
|  | Male | Female (n=62) | **-1.08** | **-2.02, -0.14** | **0.02** | **0.08** | **-1.18** | **-2.12, -0.25** | **0.01** | **0.12** |
|  |  | Male (n=80) | -0.27 | -2.07, 1.54 | 0.77 | 0.00 | 0.04 | -1.89, 1.98 | 0.97 | 0.03 |
|  |  | All (n=142) | -0.78 | -1.66, 0.09 | 0.08 | 0.02 | **-0.91** | **-1.80, -0.01** | **0.05** | **0.04** |
|  | Female | Female (n=12) | -2.99 | -7.56, 1.58 | 0.20 | 0.11 | -0.48 | -6.28, 5.32 | 0.87 | 0.26 |
|  |  | Male (n=16) | **7.13** | **1.66, 12.60** | **0.01** | **0.27** | **6.29** | **0.14, 12.45** | **0.05** | **0.31** |
|  |  | All (n=28) | 1.71 | -2.84, 6.26 | 0.46 | 0.02 | 2.82 | -1.98, 7.61 | 0.25 | 0.16 |
| ln(t-DHEA) | All | Female (n=74) | -0.12 | -0.71, 0.46 | 0.68 | 0.00 | -0.24 | -0.83, 0.36 | 0.43 | 0.03 |
|  |  | Male (n=96) | 0.18 | -0.54, 0.90 | 0.63 | 0.00 | 0.10 | -0.63, 0.84 | 0.78 | 0.04 |
|  |  | All (n=170) | -0.03 | -0.47, 0.41 | 0.88 | 0.00 | -0.09 | -0.55, 0.37 | 0.70 | 0.02 |
|  | Male | Female (n=62) | -0.13 | -0.76, 0.50 | 0.68 | 0.00 | -0.25 | -0.88, 0.38 | 0.44 | 0.04 |
|  |  | Male (n=80) | -0.03 | 0.71, 0.66 | 0.94 | 0.00 | -0.10 | -0.83, 0.64 | 0.80 | 0.03 |
|  |  | All (n=142) | -0.11 | -0.55, 0.34 | 0.64 | 0.00 | -0.15 | -0.62, 0.31 | 0.51 | 0.01 |
|  | Female | Female (n=12) | 0.33 | -1.69, 2.36 | 0.75 | 0.01 | 0.45 | -1.30, 2.21 | 0.61 | 0.28 |
|  |  | Male (n=16) | **2.73** | **0.10, 5.36** | **0.04** | **0.24** | **2.68** | **-0.45, 5.81** | **0.09** | **0.27** |
|  |  | All (n=28) | 1.03 | -0.73, 2.78 | 0.25 | 0.05 | 1.45 | -0.36, 3.25 | 0.12 | 0.20 |

^a^All models are adjusted for maternal and gestational age. In addition to maternal and gestational age the models collapsed on sex of the infant were adjusted for infant’s sex.
